# Biased Connectivity of Brain-wide Inputs to Ventral Subiculum Output Neurons

**DOI:** 10.1016/j.celrep.2020.02.093

**Published:** 2020-03-17

**Authors:** Ryan W.S. Wee, Andrew F. MacAskill

**Affiliations:** 1 Department of Neuroscience, Physiology and Pharmacology, University College London, Gower Street, London WC1E 6BT, UK

**Keywords:** hippocampus, subiculum, rabies, long range input, optogenetics, projection specificity

## Abstract

The ventral subiculum (vS) of the mouse hippocampus coordinates diverse behaviors through heterogeneous populations of pyramidal neurons that project to multiple distinct downstream regions. Each of these populations of neurons is proposed to integrate a unique combination of thousands of local and long-range synaptic inputs, but the extent to which this occurs remains unknown. To address this, we employ monosynaptic rabies tracing to study the input-output relationship of vS neurons. Analysis of brain-wide inputs reveals quantitative input differences that could be explained by a combination of both the identity of the downstream target and the spatial location of the postsynaptic neurons within vS. These results support a model of combined topographical and output-defined connectivity of vS inputs. Overall, we reveal prominent heterogeneity in brain-wide inputs to the vS parallel output circuitry, providing a basis for the selective control of individual projections during behavior.

## Highlights

• The ventral subiculum (vS) is composed of heterogenous pyramidal neuron populations
• vS projection populations are organized topographically
• vS receives long-range synaptic input from diverse areas throughout the brain
• Connectivity depends on a neuron’s downstream target and location within vS

## Graphical Abstract

**Figure:**
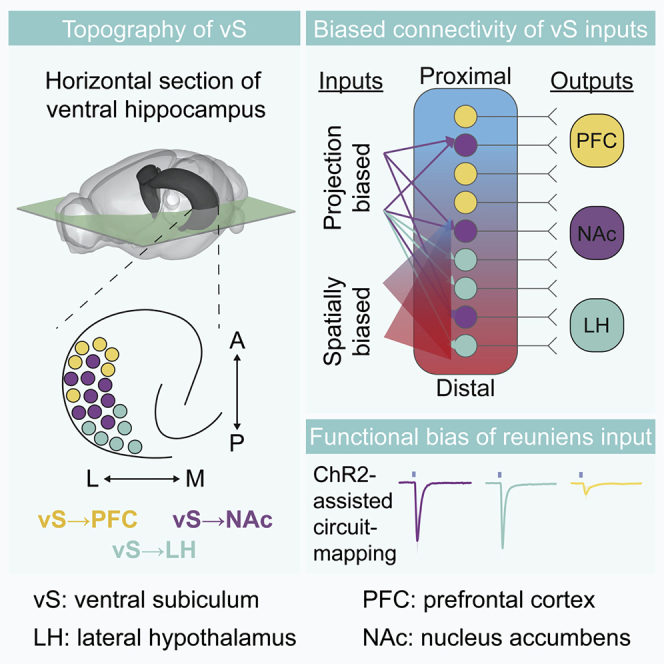

## Teaser

The ventral subiculum is formed of multiple heterogeneous populations of pyramidal neurons. Wee and MacAskill show that each of these populations receives a unique combination of long-range synaptic input. This differential connectivity may form a basis for the selective control of individual cell types during behavior.

## Introduction

The rodent hippocampus coordinates a wide array of behaviors, from spatial navigation (O’Keefe and Dostrovsky, 1971) to decision making under approach-avoidance conflict (Ciocchi et al., 2015, Jimenez et al., 2018) and reward processing (Ciocchi et al., 2015). A central hypothesis of how the hippocampus might participate in such diverse behaviors is the presence of heterogeneous principal neurons that differ widely in their gene expression (Cembrowski et al., 2016, Strange et al., 2014), electrophysiological properties (Kim and Spruston, 2012), and behavioral function (Cembrowski et al., 2018a, Cembrowski et al., 2018b, Ciocchi et al., 2015, Strange et al., 2014). In particular, the main ventral hippocampal output region, the ventral subiculum (vS), is composed of multiple neuronal populations that send parallel, long-range projections to distinct areas, including prefrontal cortex (PFC; vS^PFC^), lateral hypothalamus (LH; vS^LH^), and nucleus accumbens shell (NAc; vS^NAc^) (Naber and Witter, 1998). These populations are proposed to integrate a myriad of local and long-range inputs (Amaral and Cowan, 1980, Strange et al., 2014, Wyss et al., 1979) to perform their unique behavioral functions (Adhikari et al., 2010, Cembrowski et al., 2018a, Ciocchi et al., 2015, Jimenez et al., 2018, Soltesz and Losonczy, 2018). However, to date, knowledge of the input connectivity of vS output neurons is lacking. Further, vS populations are spatially patterned, in particular along the proximal-distal (PD) axis (ranging from the CA1 to the presubiculum borders) (Cembrowski et al., 2018a), and synaptic input varies dramatically across different spatial locations in vS (Aggleton and Christiansen, 2015, Cembrowski et al., 2018a, Knierim et al., 2013, Masurkar et al., 2017, van Groen et al., 2003). Based on this, we hypothesized that different vS output populations receive distinct upstream inputs, and we reasoned that these inputs may in turn depend on the spatial location of postsynaptic neurons (Cembrowski et al., 2016, Cembrowski et al., 2018a), their downstream target (Ciocchi et al., 2015, Jimenez et al., 2018, Naber and Witter, 1998), or a combination of these two factors.

To address this hypothesis, we studied the anatomical organization of projection-defined neurons in vS; we then applied rabies tracing across different vS projections and postsynaptic cell locations, and obtained a brain-wide map of inputs to vS subpopulations. We identified quantitative differences in multiple long-range input regions to vS that depended to different extents on the spatial location and projection target of vS neurons.

## Results

### Hippocampal Projection Populations Are Topographically Organized in vS

Each vS projection population is thought to occupy a unique spatial distribution in the hippocampus (Kim and Spruston, 2012), and long-range input into hippocampus has been shown to be highly topographical (Aggleton and Christiansen, 2015, Cembrowski et al., 2018a, Knierim et al., 2013, Masurkar et al., 2017, van Groen et al., 2003). Therefore, we first wanted to determine the spatial distribution of the different projection populations within vS, because this spatial distribution could be an important determinant for differential input connectivity.

**Figure 1:**
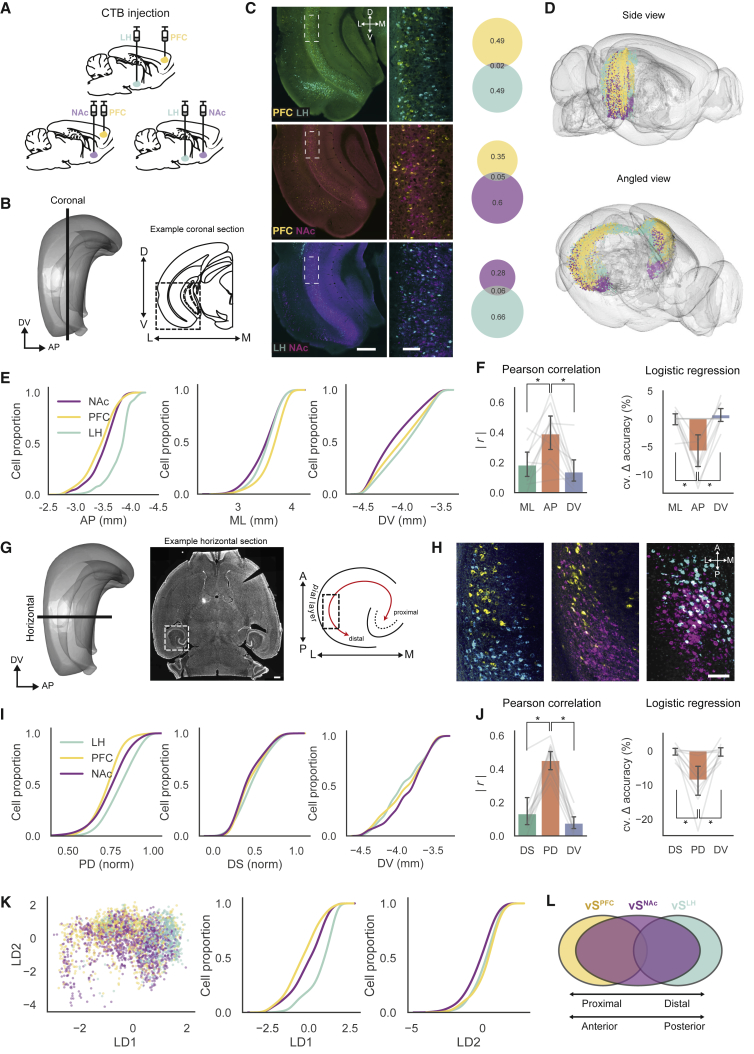
Non-overlapping vS Neurons Occupy Distinct Spatial Sites (A) Schematic of pairwise CTXβ injections. (B) Left: side view of the hippocampus with the plane of coronal sectioning illustrated. Right: example schematic of a coronal section. Boxed area is the approximate location of the example images in (C). (C) Left: examples of stitched coronal sections of vS with retrogradely labeled cells. Scale bar: 500 μm. Middle: zoom-in images of boxed area from left. Scale bar: 50 μm. Right: proportion of single- and dual-labeled hippocampal neurons. (D) 3D whole-brain diagrams with CTXβ-labeled hippocampal neurons (n = 10,8291 hippocampal neurons from 6 brains; see Video S1). (E) Cumulative distributions of CTXβ-labeled cells in ventral hippocampus along AP, ML, and DV axes (vS^LH^: n = 7,717 cells, 8 injections; vS^NAc^: n = 9,989 cells, 6 injections; vS^PFC^: n = 9,287 cells, 6 injections). (F) Left: Pearson correlation coefficient was highest along AP compared with ML and DV (n = 10 injections). Right: logistic regression analysis predicts projection type based on spatial location. Removal of AP as a predictor led to the largest decrease in accuracy (see STAR Methods). Data are summarized as mean ± SEM. Individual injections are overlaid in gray. (G) Right: side view of the hippocampus, with horizontal cutting plane illustrated. Middle: example stitched horizontal brain section. Boxed area indicates location of the left ventral hippocampus. Scale bar: 500 μm. Right: schematic of ventral hippocampus in the horizontal plane, illustrating PD axis. Boxed area is the approximate location of the example images in (H). (H) Example stitched horizontal sections of retrogradely labeled vS neurons. Scale bar: 100 μm. (I) Cumulative distributions of cell counts along the proximal-distal (PD), deep-superficial (DS), and DV axes (vS^LH^: n = 5,785 cells, 7 injections; vS^NAc^: n = 3,405 cells, 6 injections; vS^PFC^: n = 3,509 cells, 7 injections; see STAR Methods). (J) Pearson correlation (left) and logistic regression (right) analyses analogous to (F). The PD axis captures the most variation and best predicts the projection type of vS neurons (n = 10 injections). (K) LDA of registered neurons in vS unbiasedly identifies the anatomical plane that best discriminates between projection populations. Left: cell positions projected onto the first and second linear discriminant axes. Cumulative distributions of cells along the first (middle) and second (right) linear discriminant demonstrate overlap of projection populations. (L) Schematic of vS topography, where vS neurons are intermingled, vS^PFC^ neurons are located most proximally, vS^LH^ neurons most distally, and vS^NAc^ neurons span the area between both vS^PFC^ and vS^LH^. Video S1. Rotating 3D Whole-Brain View of CTXβ-Labeled Hippocampal Neurons Registered to the Allen Brain Atlas, Related to Figure 1Data from 108,921 registered, CTXβ-labeled hippocampal neurons are presented in a virtual glass brain (color code: vS^PFC^ in yellow, vS^NAc^ in magenta, vS^LH^ in cyan). Each dot represents one labeled hippocampal neuron.

To do this, we stereotaxically injected the retrograde tracer cholera toxin subunit-B (CTXβ) into PFC, LH, or NAc in a pairwise manner (Figure 1A); collected coronal sections that spanned the hippocampus; conducted whole-hippocampus cell counts; and registered the data to the Allen Brain Atlas (ABA; see STAR Methods; Figures 1B–1F). We first confirmed that vS^PFC^, vS^NAc^, and vS^LH^ neurons resided predominantly in vS (Figure 1C), and that the fraction of colocalized cells (neurons that projected to more than one injection site) ranged from 2% to 6% (Naber and Witter, 1998). Using this approach, we found that vS projection populations occupied spatially distinct locations within vS (Figures 1D and 1E). The locations of these neurons varied across all three axes (anterior-posterior [AP], medial-lateral [ML], and dorsal-ventral [DV]), but most notably along the AP axis (Figures 1E, 1F, 1H–1J), whereas vS^PFC^ neurons were located at more anterior locations, vS^LH^ neurons were located at more posterior locations, and vS^NAc^ neurons were spread across the entire range of vS. Using correlation analysis, we confirmed that projection type covaried across all axes, but most dramatically with AP position (Figure 1F), and that the spatial position of neurons along AP was most predictive of their output target (see STAR Methods; Figure 1F).

We reasoned that the marked distribution of projection populations across the AP axis might be a reflection of the known PD distribution of projection populations along the pyramidal cell layer of the hippocampus (Cembrowski et al., 2018a), which in ventral hippocampus is oriented approximately along the AP axis (Figures 1G and 1H). We confirmed that this was the case by making horizontal slices of labeled vS (Figure 1G). This experiment showed that the variance in AP is well explained by a PD gradient of neurons in vS, where vS^PFC^ neurons were located at more proximal locations near the CA1 border, vS^LH^ neurons were located at more distal locations near the presubiculum border, and vS^NAc^ neurons were spread across the entire PD axis (Figures 1H and 1I). In keeping with previous observations, correlation and regression analyses also indicated that projection type varies most along the PD compared with the deep-superficial (DS) and DV axes (Figure 1J).

Interestingly, the spatial distributions of the different projection populations were highly overlapping across each of the AP, ML, DV, and PD axes. This is notably different from that observed in dorsal subiculum (Cembrowski et al., 2018a), where there is a sharp PD border separating distinct projection populations. To control for the possibility that this sharp border was not simply obscured by our coronal or horizontal slicing angles, we carried out linear discriminant analysis (LDA) on the registered whole-hippocampus neuronal distributions (Figure 1K). This method allowed us to unbiasedly find a virtual plane that best separates the three populations of projection neurons. By examining the distribution of neurons in this plane, we observed that, although there was a clear subregion organization, the spatial distribution of the three populations in vS still remained highly overlapping (Figure 1K). Thus, in contrast with dorsal subiculum, vS appears to be organized as a gradient, especially with vS^NAc^ neurons being present at almost every point along the AP axis.

Overall, our data indicate that vS projection populations are segregated cell types and occupy overlapping yet distinct locations within vS, best separated across the AP axis.

### Labeling of Hippocampal Input Dependent on Spatial Location and Projection Target

**Figure 2:**
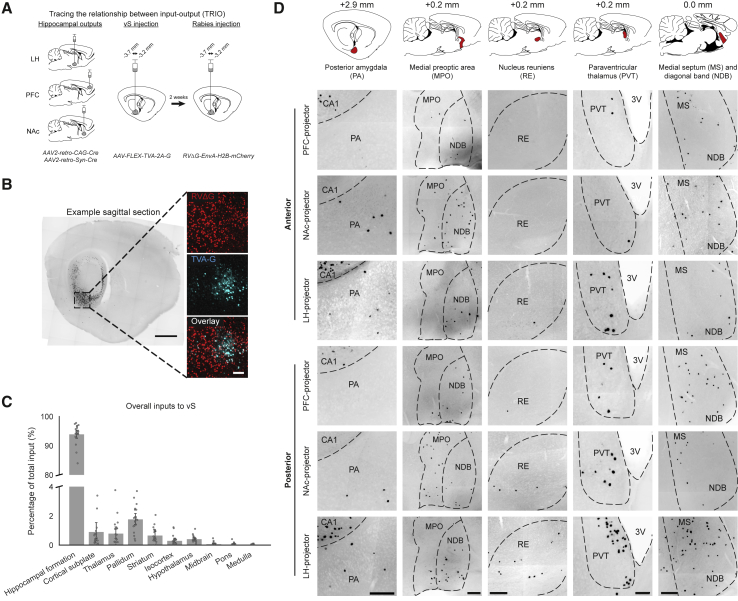
TRIO of vS Output Neurons (A) Schematic of TRIO. (B) Left: example stitched sagittal section of the brain with rabies-labeled cells (black) in the hippocampus. Right: zoom-in of boxed area from left. Starter cells colocalize for TVA-G (cyan) and rabies (red). Scale bars: 1,000 μm (right); 200 μm (left). (C) Overall input from coarse anatomical structures normalized to the total number of inputs counted in a single brain (n = 20 brains). Note the break in the y axis. Data are summarized as mean ± SEM. (D) Representative stitched images of long-range monosynaptic inputs to vS neurons by projection type and the center of mass (COM) of the starter cells along the AP axis. Classification of starter cells into anterior and posterior groups was based on the rank order of COM along the AP axis. Approximate sagittal planes are displayed with red-shaded boxes indicating the estimated locations of the corresponding images. For images of MPO input, three consecutive images (spanning 180 μm) were projected to produce the displayed representative images. 3V, third ventricle; ac, anterior commissure. Scale bars: 200 μm (all input regions except PVT); 100 μm (PVT). See also Figure S1.

Next, to directly assess the organization of presynaptic inputs onto these vS projections, we applied *tracing the relationship between input-output* (TRIO) (Beier et al., 2015, Beier et al., 2019, Ren et al., 2018, Schwarz et al., 2015) to vS projections (Figure 2A; Figures S1A–S1E). This approach involved injecting *AAV2-retro-Cre* (Tervo et al., 2016) into the output target region to retrogradely express Cre recombinase in vS neurons that projected to the target site. In the same surgery, we injected a single Cre-dependent helper construct (*AA2/1-synP-FLEX-split-TVA-2A-B19G* [TVA-G]) into vS. After at least 2 weeks of TVA-G expression in projection-specific neurons, G-deleted, pseudotyped rabies virus (*EnvA*-*RVΔG-H2B-mCherry*) harboring nuclear-localized mCherry was injected into vS to infect TVA-G^+^ cells.

Importantly, we systematically varied the injection site of TVA-G and rabies within vS (Figure 2A). This method ensured that for each projection target, we sampled distributions of starter cells (i.e., cells from which rabies virus begins the monosynaptic retrograde tracing) from different locations within vS (Figures 2A and 2B; Figure S1F). This provided us with experimental control over both starter cell location (by injection location of TVA-G and rabies within vS) and output projection (by injection location of *AAV2-retro-Cre* in target sites). This approach allowed us to assess how variations in spatial position of vS neurons (across the AP, ML, and DV axes), projection target (across PFC, NAc, and LH), and their combination influence input size and identity. In parallel, we performed control experiments where either Cre, TVA-G, or both Cre and TVA-G were excluded, to show that input labeling by rabies infection required the presence of both Cre and TVA-G (Figures S1G–S1K).

### Brain-wide Rabies Tracing Reveals Biased Connectivity of vS Projection Neurons

To quantify input to vS, we conducted brain-wide cell counts of rabies-labeled neurons in sagittal sections and registered the data to the ABA (Fürth et al., 2018, Oh et al., 2014). Consistent with previous studies, the majority (∼90%) of direct inputs to all vS projection types and spatial locations arose locally within the hippocampal formation (Figure 2C; Figures S2A–S2E). In addition, there were numerous long-range inputs arising from ∼35 brain regions spanning thalamic, striatal, pallidal, cortical, hypothalamic, and amygdalar regions (Figures 2C and 2D; Figure S2E), including the nucleus of diagonal band, medial septum, nucleus reuniens (RE), posterior amygdala (PA), and preoptic area. Further, we observed that there were variations in labeling from these regions when we compared tracing from different projection targets or starter cell position (center of mass [COM]) along the AP axis (Figure 2D).

**Figure 3:**
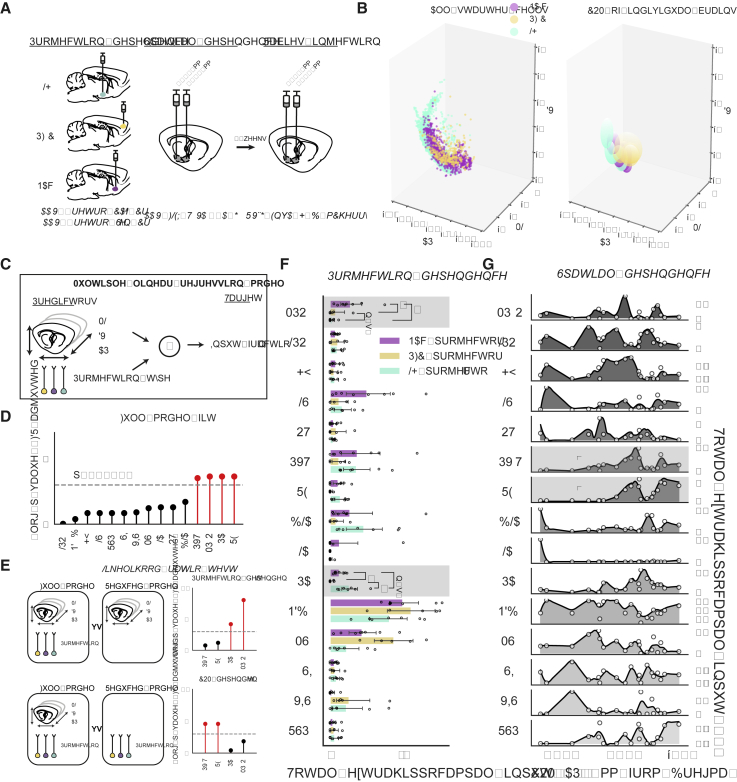
TRIO of vS Output Neurons Reveals Projection and Spatial Bias of Inputs (A) Schematic of TRIO protocol for assessing projection (via retro-Cre injection in the output site) and spatial dependence (via varying AP injection sites of TVA-G and rabies) of inputs. (B) 3D scatterplots (left) and geometric COM of individual brain samples (right) of starter cells. COM of individual brains are represented as ellipsoids (center defined as the mean position and radii as 1 standard deviation of the cell distribution in AP, ML, and DV). The spatial distribution of starter cells along the three brain axes was comparable (standard deviation of COM: AP = 0.23 mm; ML = 0.19 mm; DV = 0.28 mm). (C) Schematic of linear regression analysis. Linear models were constructed with COM (in all three brain axes), and output type as independent variables and the extrahippocampal input fraction (log-transformed) as the dependent variable (n = 20 brains). (D) Full model fits assessed by computing the F-statistic of full models with COM and projection as predictors. Inputs from PVT, MPO, PA, and RE (red) exceed the threshold of p = 0.05 after correction for multiple comparisons. (E) Likelihood ratio tests (see STAR Methods) show that MPO and PA inputs are projection dependent, whereas PVT and RE are spatially dependent. p values have been corrected for all 15 input regions tested, but only the significant hits shown in (D) are illustrated. Dashed lines indicate p = 0.05. (F) Quantification of extrahippocampal inputs. The number of inputs from a brain region is expressed as a percentage of the total number of extrahippocampal inputs (vS^NAc^: n = 8 brains; vS^PFC^: n = 5 brains; vS^LH^: n = 7 brains). Shaded boxes indicate projection-dependent input regions with significant model fits, followed up with post hoc pairwise Tukey multiple comparisons (^∗^p < 0.05). See also Video S2. Data are summarized as mean ± SEM. (G) Same dataset as in (E) where the input fractions are plotted as a function of COM along the AP axis. Shaded continuous line represents smoothed input density. Inputs from RE and PVT (∗) resulted in a significant model fit with a statistically significant COM AP coefficient. See also Figures S2 and S3 and raw cell count data in Tables S1 and S2. Video S2. Rotating 3D Whole-Brain Views of Rabies-Labeled Inputs Registered to the Allen Brain Atlas, Related to Figure 3Example data for each condition (vS^PFC^, vS^NAc^, and vS^LH^ TRIO in posterior and anterior locations) illustrated as rotating virtual glass brains. Each dot represents one rabies-labeled input neuron.

Next, we wanted to quantitatively investigate whether long-range input onto vS neurons depended on either projection target (PFC, LH, or NAc; Figure 3A), spatial location (COM in AP, ML, and DV; Figures 3A and 3B), or their combination (see STAR Methods; Figure 3). For each brain region that contributed more than 1% of extrahippocampal input, we built a linear regression model with both COM and projection information as predictors (Figure 3C) and tested the ability of the model to predict the percentage of total extrahippocampal input from that region. Using this strategy, we found that input from medial preoptic area (MPO), PA, RE, and paraventricular thalamus (PVT) provided quantitatively different input sizes to vS depending on either COM, output projection, or both predictors (Figure 3D). Importantly, these results were independent of normalization method, because we found similar projection and spatial dependence using alternative normalization procedures (Figures S3A and S3B).

We next sought to directly investigate the relative contribution of projection type or COM on the amount of RE, PVT, MPO, and PA inputs. To do this, we compared single-predictor (projection or COM) models with combined (projection and COM) models (see STAR Methods; Figure 3E). Using this approach, we found that MPO and PA innervated vS dependent solely on the projection target of the postsynaptic neuron, whereas RE and PVT inputs were dependent on COM (Figure 3E). Utilizing a similar approach, we next investigated whether AP, ML, or DV information was important for the spatial dependence of PVT and RE input, and found that for both RE and PVT, input was most dependent on starter cell location along the AP axis (Figure S3C), consistent with our predictions from Figure 1. Post hoc testing revealed that MPO input selectively innervated vS^NAc^ neurons, and PA input selectively targeted vS^LH^ and vS^NAc^ neurons, whereas RE and PVT input targeted vS^LH^ and vS^NAc^ neurons only at posterior locations within vS (Figures 3F and 3G). Subsequently, we obtained a qualitative description of all extrahippocampal inputs (35 regions) and their relative dependence on COM or projection, which revealed a wide range in the relative dependence of different synaptic input on both COM and projection identity (Figures S3D and S3E). Finally, we confirmed the spatial predictions of the rabies tracing data by analyzing anterograde labeling experiments available publicly from the ABA (Figures S3F and S3G), which showed strong spatial dependence of axon innervation across the AP axis for RE and PVT, but not for MPO or PA input.

Overall, our rabies tracing dataset identified brain-wide regions that project to vS, including quantitatively biased inputs from MPO, RE, PVT, and PA that depend differentially on the location and projection identity of the postsynaptic neuron.

### Biased Nucleus RE Input to Hippocampal Projection Neurons

**Figure 4:**
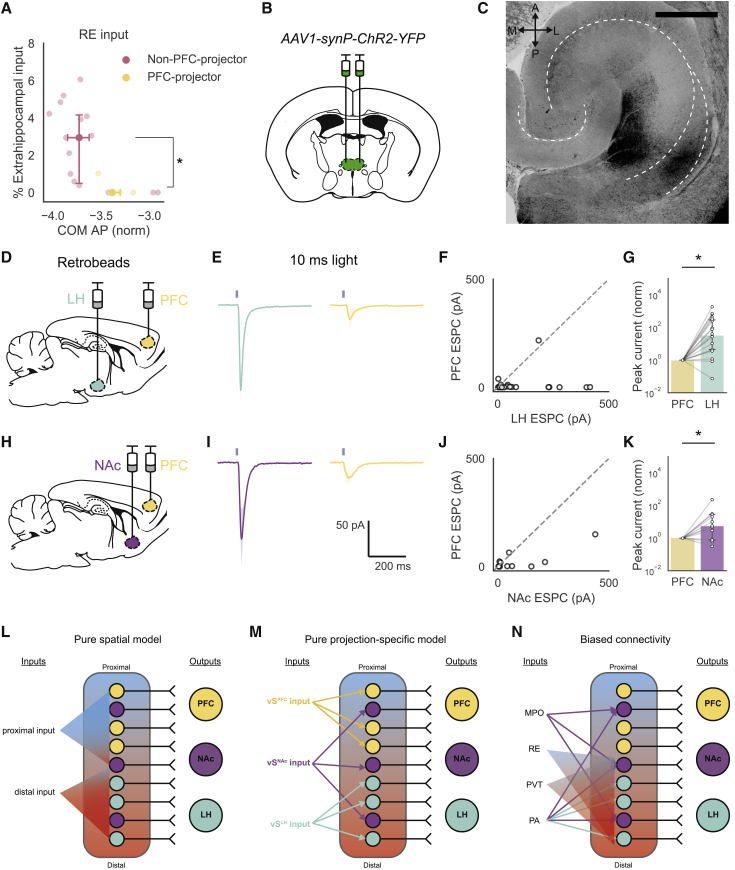
Nucleus Reuniens Inputs to vS Projection Neurons Are Functionally Biased (A) Extrahippocampal fractions of RE inputs as a function of COM and projection population. Non-PFC projectors (i.e., pooled vS^NAc^ and vS^LH^) have overall higher RE inputs than vS^PFC^ (n = 15 non-PFC projectors, n = 5 PFC-projectors; Mann-Whitney U test, U = 12.0, p = 0.013). Data are summarized as median and interquartile range. (B) AAV expressing ChR2 under the *synapsin* promoter was injected bilaterally into RE. (C) Stitched confocal image of a horizontal section of hippocampus. RE axons expressing ChR2 target distal regions of vS. Scale bar: 500 μm. (D and H) In the same surgery after ChR2 injection into RE, red and green retrobeads were injected into either LH and PFC (D) or NAc and PFC (H). (E and I) Light-evoked excitatory postsynaptic currents in pairs of (E) vS^LH^ and vS^PFC^ or (I) vS^NAc^ and vS^PFC^. All data represent responses to 10 ms of blue light. Solid line: mean response; shaded region: 95% confidence interval. (F and J) Scatterplots of light-evoked photocurrents from vS^LH^ and vS^PFC^ cell pairs (F) and vS^NAc^ and vS^PFC^ cell pairs (J). (G and K) Normalized EPSCs from vS^LH^ (G) and vS^NAc^ (K) neurons scaled to the photocurrent elicited in neighboring vS^PFC^ neurons; the relative amplitudes of light-evoked photocurrents are higher in vS^LH^ (Wilcoxon signed-rank test, n = 18 pairs from 3 animals, V = 4.0, p = 3.86 × 10^−4^) and vS^NAc^ (Wilcoxon signed-rank test, n = 11 pairs from 3 animals, V = 6.0, p = 0.016). Data are summarized as median ± 95% confidence intervals. (L–N) Models of vS input connectivity based purely on (L) spatial location or (M) projection identity. (N) Our dataset supports a combined model from (L) and (M), where inputs can be either projection or COM dependent. See also Figure S4.

A surprising finding in our dataset was that RE input was anatomically biased to avoid vS^PFC^ neurons (Figure 4A). This finding runs counter to classic models of the vS-PFC-RE-vS circuit where RE functions as a relay between PFC and hippocampus via long-range input to vS^PFC^ projections (Dolleman-van der Weel et al., 2019, Vertes, 2006). We thus sought to confirm these anatomical data using *channelrhodopsin-assisted circuit mapping* (CRACM) to ensure that this result was not due to methodological constraints such as viral tropism (Luo et al., 2018) or activity dependence of viral tracing (Beier et al., 2017) (see Discussion). From our tracing data, we hypothesized that RE input was spatially biased, i.e., RE input targets posterior areas where vS^PFC^ neurons are not abundant (COM). In addition, we wanted to ask whether RE input does not form synaptic connections even with less abundant vS^PFC^ neurons in the most posterior locations (projection).

We injected adeno-associated virus (AAV) to express ChR2 in RE (Figure 4B) and found that ChR2^+^ axons emanating from RE were observed most densely in the distal, posterior region of vS (Figure 4C), consistent with the spatial dependence of our rabies tracing data. In these slices, we recorded light-evoked postsynaptic currents from pairs of retrogradely labeled neurons within axon-rich distal vS. By recording from pairs of neighboring neurons within the same slice in the presence of tetrodotoxin (TTX) and 4-aminopyridine (4-AP), this approach allowed us to directly compare the relative monosynaptic RE input strength across projection populations while controlling for PD and AP position, ChR2 expression, and light intensity. We observed that excitatory RE input was indeed much weaker onto vS^PFC^ neurons, whereas it strongly targeted neighboring vS^LH^ and vS^NAc^ neurons (Figures 4D–4K; Figure S4). Overall, these results demonstrate a functional bias of RE inputs away from vS^PFC^ and toward both vS^LH^ and vS^NAc^.

## Discussion

Using a combination of retrograde tracing, conditional rabies tracing, and optogenetics with whole-cell electrophysiology, we demonstrated that the vS output circuitry is composed of projection-specific, topographically organized populations that receive a range of local and long-range inputs. In turn, the targeting of these inputs depends to different degrees on the spatial position and projection target of the postsynaptic vS neuron.

### Topography of vS Projections

We used CTXβ retrograde tracing to reveal the distribution of neurons in vS that project to PFC, NAc, and LH, and confirmed previous findings that suggest that there are unique populations of neurons in vS that project to each downstream region (Naber and Witter, 1998). However, due to the efficiency of retrograde labeling, significant proportions of neurons projecting to multiple downstream sites cannot be definitively ruled out. Using single-neuron tracing, it was recently shown that many neurons in dorsal subiculum do indeed project to multiple downstream areas (Winnubst et al., 2019). In the future, it will be interesting to look at individual neurons in vS and directly compare the extent of projection specificity and how this varies with spatial location. Our data, however, show that a large proportion of neurons in vS appear to project to unique downstream areas.

Consistent with previous findings, we found that different vS projections are organized topographically. vS^PFC^ neurons were located most anteriorly (along AP), vS^LH^ were found posteriorly, and vS^NAc^ were widely distributed throughout vS (Kim and Spruston, 2012, Cembrowski et al., 2018a). We demonstrated that this AP gradient is most likely due to the previously described divergence along the PD axis of the pyramidal cell layer. The spatial variation of projection neurons within vS prompted us to experimentally vary our injection coordinates during rabies tracing, which enabled us to sample starter cells from different COM positions (Figure 3B). Thus, we were able to investigate the relative contribution of both projection identity and spatial location of vS neurons on the type and amount of rabies-labeled inputs.

### Biased Input to vS Based on Projection Identity and Spatial Location

Rabies tracing identified many inputs to vS, including MPO, PA, RE, and PVT, that targeted distinct populations in vS differentially; MPO selectively innervated vS^NAc^, PA selectively innervated vS^NAc^ and vS^LH^, and RE and PVT innervated vS^NAc^ and vS^LH^ only in posterior vS. Notably, the consistent MPO inputs to vS^NAc^ have not, to our knowledge, been previously described in the literature (Wyss et al., 1979, Bienkowski et al., 2018). The specificity in input labeling across the different vS projections raises interesting questions regarding the function of these upstream neurons. For example, vS is important for social memory, and this behavior may be controlled in part by MPO input to vS^NAc^ (McHenry et al., 2017, Okuyama et al., 2016).

More generally, the rabies tracing data support a model of combined topographical and output-defined connectivity of vS inputs (Figures 4L–4N) where, depending on the upstream region, vS inputs are biased according to the location, projection type, or both of these attributes. This variation in inputs across space and projection type is in keeping with the known spatial and projection-specific functions of subiculum; across PD subdivisions, proximal vS is involved in sensory encoding (Knierim et al., 2013) and distal vS supports path integration (Cembrowski et al., 2018a, Knierim et al., 2013), whereas across projection populations, vS^PFC^ and vS^LH^ encode innate threat (Adhikari et al., 2010, Ciocchi et al., 2015, Jimenez et al., 2018) and vS^NAc^ encodes social memory (Okuyama et al., 2016). Crucially, the fact that RE inputs target vS^NAc^ and vS^LH^ neurons in posterior vS also support the existence of spatial- and projection-specific function. For example, goal-directed locomotion has been proposed to be specific to both vS^NAc^ neurons and distal subiculum (Cembrowski et al., 2018a, Ciocchi et al., 2015, Okuyama et al., 2016).

### Biased Connectivity of RE Input Away from vS^PFC^ Neurons

A surprising result from our dataset was that the RE input did not innervate vS^PFC^ neurons. RE is essential for bidirectional communication between hippocampus and PFC. This thalamic region is proposed to form an anatomical link between hippocampus and PFC, thereby closing a PFC-RE-vS-PFC loop (Dolleman-van der Weel et al., 2019, Ito et al., 2015, Vertes, 2006). Little information is present about the extent to which the projection-defined vS populations are involved in this circuit loop, and it is generally assumed that RE input is integrated by vS^PFC^ neurons to relay signals from hippocampus to PFC. We found that vS^PFC^ neurons receive few and functionally weak RE inputs. Thus, although vS^PFC^ neurons do indeed receive input from RE, our data suggest that the main effect of this input is by integration upstream of vS^PFC^ neurons (i.e., through dorsal hippocampus, entorhinal cortex, local interneurons, or other pyramidal populations) before being transmitted back to PFC. It will be necessary for future work to investigate in more detail the hippocampal microcircuitry that is involved in integrating RE input, the details of other multi-synaptic routes that may allow reciprocal connectivity between the hippocampus and PFC, and how this circuit organization contributes to behavior.

Interestingly, we also show that RE input is anatomically and functionally biased toward vS^NAc^ and vS^LH^ neurons. This observed circuit connectivity complements previous data that demonstrated key roles for RE in goal-directed planning (Ito et al., 2015) and fear generalization (Xu and Südhof, 2013), functions that may be crucial for the proposed roles of vS^NAc^ in reward seeking and vS^LH^ in anxiety (Cembrowski et al., 2018a, Ciocchi et al., 2015, Jimenez et al., 2018). Therefore, it will be important to elucidate the role of RE input in these vS-related behaviors, especially given the key role of this circuit in preclinical models of disorders such as schizophrenia, depression, and Alzheimer’s disease (Dolleman-van der Weel et al., 2019, Ito et al., 2015, Vertes, 2006).

### Limitations of the Study

Although conditional rabies tracing is now a widely used technique that enables systematic brain-wide mapping of synaptic input (Beier et al., 2015, Beier et al., 2019, Do et al., 2016, Ren et al., 2018, Ährlund-Richter et al., 2019, Sun et al., 2019), it is important to note the many caveats to this technique when considering the biological meaning of such tracing data. For instance, the mechanism of transsynaptic retrograde spread is almost completely unknown (Luo et al., 2018). This raises potential confounds; for example, the efficiency of rabies viral spread may depend on uncontrolled variables such as tropism for certain cell types and synapses (Luo et al., 2018), the level of upstream circuit activity (Beier et al., 2017), or even non-synaptic transfer (Luo et al., 2018). In addition, there is conflicting evidence as to whether the quantity of rabies-labeled input neurons correlates with functional synaptic strength (Luo et al., 2018). Although the number of input neurons has been shown to match the connection probability and synaptic strength in multiple studies across many brain regions (Lerner et al., 2015, Sun et al., 2014, Kohara et al., 2014), other studies did not reach similar conclusions and showed marked divergence (Smith et al., 2016, Choi et al., 2019).

To mitigate the potential shortcomings of the technique, it is therefore important to conduct complementary experiments and analyses. One method is to combine findings from rabies tracing experiments with analysis of publicly available anterograde tracing experiments, such as those from the ABA Mouse Connectivity Atlas (Beier et al., 2019). For example, we used this approach to confirm direct input to vS from MPO, and also the spatial targeting of RE and PVT input (Figure S3). A second, more powerful method is to combine tracing with follow-up experiments that directly address synaptic strength, such as CRACM (Lerner et al., 2015). We used this approach to confirm that the RE input is stronger at synapses onto vS^NAc^ and vS^LH^ and weaker onto vS^PFC^ (Figure 4). In this instance, the functional strength of input correlated with the number of rabies-labeled inputs, but this is not always guaranteed.

Finally, there are other technical considerations that are important to highlight with the use of whole-brain mapping. First, the plane of sectioning is an important consideration and should be motivated by the purpose of the experiment. This is because there is potential for reduced sampling resolution across the axis of slicing, although error associated with this was recently shown to be minimal when detailed registration is carried out (Fürth et al., 2018). In our study, we used sagittal sectioning in order to allow accurate estimates of AP position within the brain (Wall et al., 2013, Roy et al., 2017, Beier et al., 2011), but both horizontal and coronal sections have also been repeatedly and successfully used (Bienkowski et al., 2018, Cembrowski et al., 2018a, Sun et al., 2014, Beier et al., 2015, Weissbourd et al., 2014). In addition, the use of stereotaxic injections means that despite small injection volume and controlled rate of injection, we cannot completely exclude the possibility that small amounts of virus or tracer might have leaked into neighboring regions. Finally, the success of tracing experiments is dependent on the efficiency of the tracing system, and novel variants of the rabies system, such as more efficient glycoproteins (Kim et al., 2016), or more stable rabies virus (Reardon et al., 2016, Ciabatti et al., 2017, Chatterjee et al., 2018) could enhance the power of whole-brain anatomy experiments in the future.

Overall, our study has revealed a basis for the selective control of vS projection neurons through the biased organization of brain-wide input connectivity. Further work will be required to comprehensively delineate the functional connectivity and behavioral relevance of these dedicated circuits in the execution of adaptive behavior.

## STAR★Methods

### Key Resources Table

**Table:** 

REAGENT or RESOURCE	SOURCE	IDENTIFIER
**Bacterial and Virus Strains**		

*pAAV2-retro-CAG-Cre*	Tervo et al., 2016, UNC Vector Core	N/A
*pENN-AAV-hSyn-Cre-WPRE-hGH*	a gift from James M. Wilson	Addgene viral prep # 105553-AAV1; http://addgene.org/105553; RRID:Addgene_105553
*pAAV-synP-FLEX-splitTVA-EGFP-B19G*	a gift from Ian Wickersham	Addgene viral prep # 52473-AAV1; http://addgene.org/52473; RRID:Addgene_52473
*RabiesΔG-EnvA-H2B-mCherry-2A-CLIP*	a gift from Marco Tripodi, MRC LMB	N/A
*pAAV-hSyn-hChR2(H134R)-EYFP*	a gift from Karl Deisseroth	Addgene viral prep # 26973-AAV1; http://addgene.org/26973; RRID:Addgene_26973

**Chemicals, Peptides, and Recombinant Proteins**		

Red retrobeads	Lumafluor	Item #: R180
Green retrobeads	Lumafluor	Item #: G180
Cholera Toxin Subunit B (Recombinant), Alexa Fluor 647 Conjugate	Invitrogen	Cat #C34778
Cholera Toxin Subunit B (Recombinant), Alexa Fluor 555 Conjugate	Invitrogen	Cat #C34776
ProLong Gold Antifade Mountant with DAPI	Invitrogen	Cat #P36930
ProLong Glass Antifade Mountant with DAPI	Invitrogen	Cat #P36984
Isoflurane	Piramal Critical Care	
Carprofen	Norbrook	
Tetrodotoxin	Hello Bio	Cat #HB1035
4-aminopyridine	Hello Bio	Cat #HB1073

**Experimental Models: Organisms/Strains**		

Mouse: C57BL/6	Charles River	N/A

**Software and Algorithms**		

ImageJ (Fiji) Software	https://fiji.sc/	N/A
Python 3.6	https://www.python.org/	N/A
Jupyter Notebook	https://jupyter.org/	N/A
R	https://www.r-project.org/	N/A
WholeBrain	http://www.wholebrainsoftware.org/Fürth et al., 2018	N/A

### Lead Contact and Materials Availability

Further information and requests for resources and reagents should be directed to and will be fulfilled by the Lead Contact, Andrew MacAskill (a.macaskill@ucl.ac.uk). This study did not generate new unique reagents.

### Experimental Model and Subject Details

#### Mice

Young adult C57BL/6 male mice (CTXβ and rabies tracing: at least 7 weeks old; physiology: 7 – 9 weeks old) provided by Charles River were used for all experiments. All animals were housed in cages of 2 to 4 in a temperature- and humidity-controlled environment with a 12 h light-dark cycle (lights on at 7 am to 7 pm). Food and water were provided *ad libitum*. All experiments were approved by the UK Home Office as defined by the Animals (Scientific Procedures) Act, and strictly followed University College London ethical guidelines.

### Method Details

#### Stereotaxic surgery

Stereotaxic surgeries were performed according to previously described protocols (Cetin et al., 2006). Mice were anaesthetised with isoflurane (4% induction, 1.5 to 2% maintenance) and secured onto a stereotaxic apparatus (Kopf). A single incision was made along the midline to reveal the skull. AP, ML and DV were measured relative to bregma, and craniotomies were drilled over the injection sites. Long-shaft borosilicate pipettes were pulled and backfilled with mineral oil, and viruses were loaded into the pipettes. Viruses were injected with a Nanoject II (Drummond Scientific) at a rate of 13.8 nL every 10 s. Following infusion of the virus, the pipette was left in place for an additional 10 mins before being slowly retracted. The following coordinates (in mm) were used:

**Table:** 

Injection site	ML	AP	DV
Medial prefrontal cortex:	0.4	+2.3	−2.4
Lateral hypothalamus:	0.9	−1.3	−5.2
Nucleus accumbens (medial shell):	0.9	+1.1	−4.6
Ventral subiculum (anterior):	3.4	−3.2	−4.3
Ventral subiculum (posterior):	3.4	−3.7	−4.3
Nucleus reuniens	0.25	−0.7	−4.4

Following injection of substances into the brain, animals were sutured and recovered for 30 mins on a heat pad. Animals received carprofen as a peri-operative s.c. injection (0.5 mg / kg) and in their drinking water (0.05 mg / mL) for 48 hours post-surgery.

The titers of viruses used are the following:

• *RabiesΔG-EnvA-H2B-mCherry-2A-CLIP,* 1.8 × 10^8^ genome copies (gc) / mL;
• pAAV-synP-FLEX-splitTVA-EGFP-B19G, 3.9 × 10^12^ gc / mL;
• pENN-AAV-hSyn-Cre-WPRE-hGH, 1.3 × 10^13^ gc / mL;
• pAAV-hSyn-hChR2(H134R)-EYFP, 2.5 × 10^13^ gc / mL;
• pAAV2-retro-CAG-Cre, 5.3 × 10^12^ gc / mL

#### Retrograde tracing

For CTXβ retrograde tracing, 150 nL of Alexa 555- or 647-tagged CTXβ was injected into one of three output regions (PFC, NAc or LH). After at least 14 days post-surgery, animals were sacrificed for histology. For rabies monosynaptic tracing, experiments were done according to previously described protocols (Beier et al., 2015). Adult male mice were injected with 100 nL of *AAV2-retro-CAG-Cre* or *AAV2-retro-synP-Cre* into one of three output regions (PFC, NAc or LH), and in the same surgery, 250 nL of *AA2/1-synP-FLEX-split-TVA-EGFP-B19G* was injected into ventral subiculum (anterior) or ventral subiculum (posterior). In a second surgery after at least 2 weeks, 300 – 400 nL of *EnvA-RVΔG-H2B-mCherry* was injected into vS. After 7 days of rabies expression, animals were sacrificed for histology.

#### Histology and imaging

Animals were deeply anaesthetised with a lethal dose of ketamine and xylazine (100 mg/kg) and perfused transcardially with phosphate-buffered saline (pH = 7.2) followed by 4% paraformaldehyde. Brains were dissected and post-fixed overnight at 4°C prior to sectioning. For CTXβ tracing analysis, 60-μm sections in the horizontal plane were prepared using a vibratome (Campden Instruments). For brain-wide rabies tracing analysis, 60-μm sections in the sagittal plane were prepared with a supporting block of agar, and every 2^nd^ section was mounted and analyzed. Sections were mounted on Superfrost Plus slides with ProLong Gold or ProLong Glass antifade mounting medium (Molecular Probes) and imaged with a 5x objective using a Zeiss Axioscan Z1 or 10x objective using a Zeiss SLM 800, using standard filter sets for excitation/emission. Tiled images were stitched automatically during image acquisition by Zen software to generate the full images displayed.

#### Whole-hippocampus cell quantification and analysis

For quantification of CTXβ-labeled hippocampal neurons, consecutive 60-μm coronal sections spanning the hippocampus (approximately AP −4.3 mm to −1.0 mm) were collected. Cell counting of retrogradely labeled neurons was conducted using custom written scripts in R based around the WholeBrain package (Fürth et al., 2018), a recently developed automatic segmentation and registration workflow in R. Only sections containing labeled neurons in the hippocampus (∼-4.1 mm to −2.7 mm) were analyzed. Segmentation was performed using wavelet multiresolution decomposition on the WholeBrain platform in R, and the segmentation parameters (pixel threshold, soma size, brain outline etc.) were adjusted slice by slice to achieve accurate segmentation of neurons. For the registration of coronal sections, six to eight random brain sections were sampled and manually annotated with approximate AP coordinates, and the remaining sections were assigned AP coordinates based on interpolation. For a given experiment, two projection-specific hippocampal populations (pairs of NAc, LH or PFC) were labeled with two fluorophores (Alexa Fluor 555 and Alexa Fluor 647) in a counterbalanced order. First, segmentation was conducted for each channel individually. Subsequently, registration was conducted on one image data from one channel in a semi-automated manner; this involved an automatic registration of the tissue section by the WholeBrain software, and then refining the registration through manually adding, subtracting or changing correspondence points at clear anatomical landmarks. The same anatomical registration was applied to register the tissue section image obtained from the other channel. Cell count data were saved as RData files, imported into Python and analyzed using Python 3.6.

For spatial distribution, correlation and logistic regression analyses, individual sections were manually assigned AP coordinates by first estimating the posterior-most AP coordinate using the ABA as a reference, and then labeling the next anterior coronal section at 60-μm increments until the last section in the coronal section series. ML and DV positions were calculated through registration of individual neurons to the ABA (see above). Only hippocampal cells in subiculum (‘SUB’) and CA1 residing in ventral hippocampus (∼DV −3.5 and −4.5 mm) were analyzed. Cell distributions across AP, ML and DV were determined by kernel density estimation using a Gaussian kernel function and bandwidth estimated by Scott’s rule.

Correlation analysis was conducted to determine which brain axis covaries most with the projection type of hippocampal neurons. For each pair of injections in a given hemisphere, the covariation of spatial position along each axis with projection type was computed using Pearson correlation using the *scipy* function *scipy.stats.pointbiserialr*. The absolute value of the correlation coefficient indicates the degree of covariation, where |r| = 1 indicates perfect correlation, and |r| = 0 indicates no correlation; the absolute correlation coefficient was then compared across the different brain axes.

To identify which axis contributes most information to predict the projection class of hippocampal neurons, we conducted logistic regression analysis using the *sklearn* package in Python. For this analysis, the total ventral hippocampal cell counts from each hemisphere were divided into 80% training dataset to train the multinomial logistic regression model, and 20% test dataset to examine the performance of the model. The train-test-split was crucial to assess how well the linear classifiers generalized to unseen data. The 10-fold cross-validated accuracy of the models was calculated using the *LogisticCV* function, and the classifiers were assessed for how much their performance degraded after removal of each brain axis (AP, ML or DV) as a predictor in the model. This reduction in accuracy after the removal of one predictor (cv. Δ accuracy) indicates the unique information that spatial position along one axis contributes to their performance. Note that 2 hemispheres out of 12 total hemispheres from 6 animals were excluded from the dataset due to poor hippocampal labeling.

#### Analysis of spatial positions of vS projections along the PD axis

For a subset of CTXβ injected brains, brain sections were prepared in the horizontal plane to analyze the spatial positions of retrogradely labeled vS projections to PFC, NAc and LH along the PD axis. Six to eight sections spanning vS (approximately DV −3.5 to −4.5 mm) were analyzed per brain hemisphere. Using ImageJ, horizontal sections of the hippocampus were digitally straightened from the dentate gyrus to the end of subiculum using the *Straighten* function on ImageJ to approximate the PD axis. Labeled cells in each slice were manually counted and registered to this axis using the ImageJ CellCounter plugin. The radial (y-coordinate) and PD (x-coordinate) positions of each cell were used for spatial position analysis. The DV positions of each cell was manually annotated for each hippocampal slice based on the Paxinos atlas. The CA1/subiculum border occurs approximately at 0.7 within this normalized PD axis range and was anatomically defined as the disappearance of stratum oriens and the fanning out of the pyramidal cell layer. The spatial positions were analyzed with custom Python routines.

#### Linear discriminant analysis (LDA) of registered hippocampal neurons

LDA was used to unbiasedly identify the plane which most optimally separates the clusters of CTXβ-labeled, projection-specific hippocampal neurons. LDA is a dimensionality reduction method that identifies the subspace which maximizes the ratio of the between-class over the within-class variability. The between- and within-class variability were calculated as scatter matrices S_B_ and S_W_, respectively, where S_B_ and S_W_ are 3x3 matrices and the number of rows or columns corresponds to each brain axis (AP, ML and DV).The predictor variables for the LDA analysis were the registered spatial positions in AP, ML and DV, and the target variable was the projection type (encoded labels of NAc, PFC or LH). We focused on hippocampal cells labeled between DV −4.5 mm and −4.0 mm, and this dataset (n = 14,527 ventral hippocampal neurons counted from 10 experiments; 6 animals) was split into an 80% training and 20% held-out test dataset. The projection matrix used to transform the retrogradely labeled neurons to the subspace that best maximizes discriminability was solved by matrix factorisation using singular value decomposition (SVD) based on the *LinearDiscriminantAnalysis* function from the *sklearn* package. The spatial positions of the held-out test dataset were then projected onto the subspace by matrix multiplication of the spatial position and projection matrices:

X'=Xφ

where φ is the eigenvector (projection) matrix whose columns correspond to the eigenvectors (linear discriminant vectors), and *X* is the matrix of spatial positions of each registered neuron. The transformed spatial positions were plotted in the LD subspace, and the cell distributions in the first and second linear discriminants were determined by kernel density estimation.

#### Mapping and analysis of rabies-labeled inputs

Cell counting of rabies-labeled inputs was conducted using WholeBrain (Fürth et al., 2018). After acquiring the imaged sections and exporting them as 16-bit depth image files, images in the rabies mCherry channel were manually assigned a bregma coordinate (ML −4.0 to 0.0 mm) and processed using WholeBrain (Fürth et al., 2018) and custom cell counting routines written in R. The workflow comprised of (1) segmentation of cells and brain section, (2) registration of the cells to the ABA and (3) analysis of anatomically registered cells. As tissue section damage impairs the automatic registration implemented on the WholeBrain platform, sections with poor registration were manually registered to the atlas plate using corresponding points to clear anatomical landmarks. Once all cells have been registered, the cell counts were further manually filtered from the dataset to remove false-positive cells (e.g., debris). Virtually all cells were detected in the injected hemisphere, apart from a consistent set of contralateral CA3 inputs (Bienkowski et al., 2018). Therefore, only the injected hemisphere up to the midline was used for cell quantification analysis.

Each cell registered to a brain region was classified as belonging to an anatomically defined region as defined by the ABA brain structure ontology. Information on the ABA hierarchical ontology was scraped from the ABA API (link: http://api.brain-map.org/api/v2/structure_graph_download/1.json) using custom Python routines. The coarse-level (or parent) structures to which each cell belonged were defined *a priori* and comprised the following: *Hypothalamus, Isocortex, Hippocampal formation, Thalamus, Cortical subplate, Pallidum, Striatum, Midbrain, Pons* and *Medulla.* All cells falling under these parent structures were analyzed. The fine-level (or child) structures represent all brain regions existing as subcategories of the corresponding coarse-level structure, e.g., *nucleus reuniens* and *paraventricular thalamus* are fine-level (child) structures relative to *Thalamus* (parent). For quantification of input fractions, cells residing in different layers within the same structure, e.g., CA1 stratum oriens (CA1so) and stratum lacunosum-moleculare (CA1slm), or subdivisions of nuclei, e.g., basomedial amygdala, posterior division (BMAp) and anterior division (BMAa), were agglomerated across layers and subdivisions and counted as residing in one single region (CA1 and BMA, respectively). Note that lateral and medial entorhinal cortex (LEC and MEC, respectively) were analyzed as separate structures.

#### Starter cell center of mass (COM) quantification

To determine the starter cell COM, every 2^nd^ sagittal section from both the rabies mCherry+ and TVA-G GFP+ channels that spanned the extent of the TVA-G GFP+ expression in vS was obtained and analyzed. Images were collected in order from lateral to medial. Colocalized cells representing starter cells were manually counted and registered onto digital plates from the Paxinos atlas using the ImageJ ROI Manager function. Cell positions in AP and DV were calculated by first obtaining the spatial position of cells in pixel space. Then, the location of bregma in pixel space was determined by placing an ROI at the approximate site of bregma in the Paxinos atlas. Finally, a scaling factor was calculated by estimating the change in pixel space with a unit change in ML and DV. The final positions of cells were calculated by offsetting the positions of the cells in pixel space to that of bregma and normalizing the positions by the scaling factor. As the TVA-G construct expressed TVA and G bicistronically (i.e., the exon for TVA and G are linked by a self-cleaving 2A peptide), all cells were assumed to express TVA and G in a 1:1 stoichiometry and unlikely to express one gene without the other. Therefore, all colocalized cells were treated as starter cells.

#### Analysis of COM versus projection dependence of inputs

Inputs arising from within the hippocampal formation were analyzed as input fractions of the total inputs counted in the same brain, while inputs arising from outside the hippocampal formation were analyzed as input fractions normalized to the total number of extrahippocampal inputs. The dataset containing cell counts from n = 20 brains were analyzed according to projection or COM. For extrahippocampal inputs, only fine-level structures exceeding 1% of extrahippocampal input were assessed (15 brain regions).

Multiple linear regression modeling was performed to compare the relative influence of COM and projection identity on the amount of rabies-labeled inputs in an input region of interest. For each input brain region, a multiple regression model – in which the predictor variables were the COM and projection identity – was constructed using the *ols* function from the *statsmodel* package in Python. The overall statistical significance of full models (containing COM and projection as predictors) were assessed by computing the F-statistic values. All *p* values generated from multiple comparisons were corrected using the Benjamini-Hochberg method (false-discovery rate < 0.05). The models with adjusted p values < 0.05 were further analyzed for statistically significant coefficients using the Wald test and followed up with post hoc pairwise Tukey multiple comparisons between vS projection populations (see Figures S3C and 3F).

To further assess the importance of each predictor to the model, the likelihood ratio (LR) test was used to compare the full model to a reduced model containing only one predictor – either COM or projection. The reduced models containing either COM or projection identity as a predictor were built using the same *ols* function. The LR test was computed using the following formula from the *statsmodel* package function *compare_lr_test:*

$$D=-2\;\log\left(\frac{L_{restricted}}{L_{full}}\right)$$

where L is the likelihood of the model, and D is a test statistic that follows a $\chi^{2}$ distribution with degrees of freedom (df) equal to the difference in the number of predictors between the full and restricted (reduced) model. The p values generated from multiple LR tests were corrected using the Benjamini-Hochberg method (FDR < 0.05). As complementary analyses, the projection-dependence of extrahippocampal inputs from Figure 3F was further analyzed using multiple one-way ANOVAs, while the spatial-dependence of these inputs from Figure 3G was assessed using multiple Spearman rank correlation tests of input fractions against starter cell COM. This analysis revealed similar patterns of biased connectivity to the multiple linear regression analysis (Table S2), where MPO and PA were detected as projection-dependent inputs, but RE was detected as being COM-dependent.

For spatial-dependence analysis, the input density for each brain region as a function of COM was visualized by first sorting the input fractions of each brain by the COM in the posterior-to-anterior direction. The array of input fractions was then interpolated to produce 500 data points, smoothed with a Savitzky-Golay filter (window size = 51 points, order = 3), and normalized by dividing each data point by the total area under the curve.

To assess the relative goodness-of-fit of non-nested *COM models* or *projection models*, we performed linear regression models with only one predictor by using the *ols* function from the Python package *statsmodels*. Linear models were built for each brain region, where the target variable was the input fraction observed in that brain region normalized to the total number of inputs, and the predictor variable was either the COM or projection. For each of 35 extrahippocampal inputs in total, there were 20 observations (n = 20 brains). The models were fitted, and goodness-of-fit was measured using the Bayesian information criterion (BIC). The BIC was computed using the following formula:

$$BIC=k\;\;\log\;n-2\;\log\hat{L}$$

where *k* is the number of model parameters, *n* is the observation number and $\hat{L}$ is the maximum likelihood of the model. Finally, to compare model fits, the difference between the BIC values obtained from COM and projection models were computed to obtain ΔBIC.

#### ABA Mouse Brain Connectivity analysis of axonal projections

To validate the spatial targeting of rabies-labeled inputs as predicted by the multiple linear regression analysis, analysis of axonal input density arising from the input region ‘hits’ (i.e., MPO, RE, PA and PVT) was conducted. For MPO, PVT and RE, three separate experiments were analyzed. For PA, the only two experiments available were analyzed. All images were downloaded from the ABA API using the Python package Allen SDK (2015). The following experiments were used:

**Table:** 

Input	Experiment ID	Transgenic line	Image sections used for analysis
MPO	158738180	*C57BL/6*	158738374, 158738370, 158738366, 158738362, 158738358, 158738354
	119846838	*C57BL/6*	119847348, 119847344, 119847340, 119847336, 119847332, 119847328
	182459635	*Gal-Cre_KI87*	182460001, 182459997, 182459993, 182459989, 182459985, 182459981
RE	204832205	*Adcyap1-2A-Cre*	204832574, 204832570, 204832566, 204832562, 204832558, 204832554
	175374982	*C57BL/6*	175375180, 175375176, 175375172, 175375168, 175375164, 175375160
	174957972	*C57BL/6*	174958286, 174958282, 174958278, 174958274, 174958270, 174958266
PA	304721447	*Rbp4-Cre_KL100*	304721806, 304721802, 304721798, 304721794, 304721790, 304721786
	545415593	*Npr3-IRES2-Cre*	545415944, 545415940, 545415936, 545415932, 545415928, 545415924
PVT	278510903	*Ppp1r17-Cre_NL146*	278511259, 278511255, 278511251, 278511247, 278511242, 278511238
	183225830	*Grm2-Cre_MR90*	183226060, 183226056, 183226052, 183226048, 183226044, 183226040
	301209502	*Efr3a-Cre_NO108*	301209844, 301209840, 301209836, 301209832, 301209828, 301209824

For each experiment, six consecutive coronal images (spanning approximately −3.7 to −3.1 mm AP relative to bregma) of ventral hippocampus were downloaded and analyzed using ImageJ. Only one hemisphere per brain was analyzed. The brightness and contrast values were fixed for all sections obtain from a single experiment to allow comparison of the relative fluorescence of axonal projections across slices. Images were downloaded with a downsample factor of 8, ROIs were manually drawn around the vS (defined as the region below the rhinal sulcus, between the alveus and the boundary of the CA1 stratum lacunosum moleculare and adjacent dentate gyrus or CA3 stratum radiatum border, see also Figures S3F and S3G), and the mean pixel intensity within the ROIs were measured. The pixel intensity values were normalized to that from the posterior-most section, and the relative fluorescence intensities were compared across the AP axis.

#### CRACM experiments

##### Surgeries

CRACM of projection-defined vS neurons was done according to previously described protocols (MacAskill et al., 2014). 7- to 9-week old animals were injected with 250 nL of red (1:10 dilution in sterile saline) or green (undiluted) retrobeads in a counterbalanced order into two of three output regions (PFC, NAc or LH). In the same surgery, 250 nL of *AAV1-synP-ChR2-YFP* was injected into RE bilaterally. After at least 14 days of ChR2 expression, animals were sacrificed for electrophysiological recording.

##### Slice preparation

Acute, transverse hippocampal slices were used for all electrophysiological recordings. Mice were deeply anaesthetised with a lethal dose of ketamine and xylazine (100 mg / kg), and perfused transcardially with ice-cold sucrose solution containing (in mM): 190 sucrose, 25 glucose, 25 NaHCO_3_, 1.2 NaH_2_PO_4_, 10 NaCl, 2.5 KCl, 1 Na^+^ ascorbate, 2 Na^+^ pyruvate, 7 MgCl_2_ and 0.5 CaCl_2,_ bubbled continuously with 95% O_2_ / 5% CO_2_. Following perfusion, mice were decapitated, and their brains were rapidly dissected. The dissected brains were then placed in ice-cold sucrose solution and hemisected. The cerebellum was removed, and transverse slices were prepared using a vibratome (VT1200S, Leica), with a ∼10° angle along the ventromedial plane to obtain sections that were orthogonal to the long-axis of the hippocampus. The thickness of hippocampal sections was 300 μm. Slices were transferred to a bath containing artificial cerebrospinal fluid (aCSF) and recovered first for 30 mins at 37°C, and subsequently for 30 mins at room temperature. The aCSF solution contained (in mM): 125 NaCl, 2.5 KCl, 1.25 NaH_2_PO_4_, 22.5 glucose, 1 Na^+^ ascorbate, 3 Na^+^ pyruvate, 1 MgCl_2_, 2 CaCl_2_. All recordings were performed at room temperature (22 – 24°C). All chemicals were from Sigma or Tocris.

##### Whole-cell electrophysiology and optogenetics

Whole-cell recordings were performed on retrogradely labeled hippocampal pyramidal neurons with retrobeads visualized by their fluorescent cell bodies and targeted with Dodt contrast microscopy. For sequential paired recordings, neighboring neurons were identified using a 40x objective at the same depth into the slice. The recording order of neuron pairs was alternated to avoid complications due to rundown. Borosilicate recording pipettes (3 – 5 MΩ) were filled with a Cs-gluconate internal solution containing (in mM): 135 Gluconic acid, 10 HEPES, 10 EGTA, 10 Na-phosphocreatine, 4 MgATP, 0.4 Na_2_GTP, 10 TEA and 2 QX-314. Presynaptic glutamate release was elicited by illuminating ChR2 expressed in the presynaptic terminals of long-range inputs into the slice, as previously described (MacAskill et al., 2014). Wide-field illumination was achieved through a 40x objective with brief 10 ms pulses of blue light over the recorded neuron from an LED centered at 473 nm (CoolLED pE-4000, with corresponding excitation-emission filters). Light intensity was measured as 4–7 mW at the back aperture of the objective and was constant between all recorded cell pairs. When recording a cell pair, the LED power was adjusted until responses were ∼200 pA in a connected neuron or set to maximum in an unconnected neuron, and fixed at the same level when recording light-evoked responses in the other cell. In all experiments, the aCSF contained 1 μM TTX and 100 μM 4-AP to isolate monosynaptic connectivity and increase presynaptic depolarisation, respectively. Recordings were conducted using a Multiclamp 700B amplifier (Axon Instruments), and signals were low-pass filtered using a Bessel filter at 1 kHz and sampled at 10 kHz. Data were acquired using National Instruments boards and WinWCP (University of Strathclyde) and analyzed using custom routines written in Python 3.6.

For synaptic connectivity analysis, six recording sweeps were obtained for each optical pulse duration. The signals were preprocessed by baselining the signals to the first 100 ms, low-pass filtering using a Bessel filter (cutoff = 2 Hz, order = 2), averaging across the six recording sweeps for each optical pulse duration, and decimating the averaged signal to 1 kHz. The peak amplitude response of light-evoked EPSCs was measured as averages over a 2-ms time window around the peak compared to a 2-ms baseline period preceding the optical pulse. Only paired data in which at least one cell received > 5 pA were included for analysis for Figures 4D–4K, while all cells irrespective of connectivity were included for the analysis described in Figure S4. We note that due to the jitter in release as a result of the use of presynaptic ChR2, across-cell averages of postsynaptic currents (Figures 4D–4K) may appear relatively slow. For each recorded neuron, the spatial position was obtained by manually registering the cell to a digital atlas depicting a horizontal section of the vS. The long axis of the CA1 and subiculum area (i.e., the y-coordinates) of each registered cell was used to approximate the anterior-posterior position. Input connectivity was determined by a threshold light-evoked response of > 5 pA.

### Quantification and Statistical Analysis

All statistics were calculated using Python’s *scipy* and *statsmodels* packages, and R. Summary data are reported throughout the text and figures as mean ± sem unless otherwise stated. For Figures 1 and 4, normality of data distributions was determined by visual inspection of the data points. For Figures 3 and S2, the rabies tracing dataset was assessed for normality with the Jarque-Bera test and equal variance with the Levene’s test. The input fractions for multiple regions showed non-normality and heteroscedasticity in the input fractions across projection populations. Therefore, all analysis for Figure 3 were conducted after log-transformation of input fractions. Distributions of the data became more Gaussian-like after log-transformation, as assessed again by the Jarque-Bera test and Levene’s test. All data were analyzed using statistical tests described in the figure legends and in Table S2. The alpha level was defined as 0.05. No power analysis was run to determine sample size *a priori*. The sample sizes chosen are similar to those used in previous publications.

### Data and Code Availability

Programme code, original data and scripts for statistical tests and analysis are available upon request. Raw data are available at https://github.com/macaskill-lab.

## Supplemental Information

Document S1. Figures S1–S4 and Tables S1 and S2

Data S1. Raw Cell Counts in Each Condition of the TRIO Experiments (vS^PFC^, vS^NAc^, and vS^LH^ Projections with Associated COM Information), Related to Figure 3

Data S2. Abbreviations of the 115 Brain Regions with Raw Cell Counts Shown in Data S1, Related to Figure 3

Document S2. Article plus Supplemental Information

## References

[bib1] Adhikari A., Topiwala M.A., Gordon J.A. (2010). Synchronized activity between the ventral hippocampus and the medial prefrontal cortex during anxiety. Neuron.

[bib2] Aggleton J.P., Christiansen K. (2015). The subiculum: the heart of the extended hippocampal system. Prog. Brain Res..

[bib3] Ährlund-Richter S., Xuan Y., van Lunteren J.A., Kim H., Ortiz C., Pollak Dorocic I., Meletis K., Carlén M. (2019). A whole-brain atlas of monosynaptic input targeting four different cell types in the medial prefrontal cortex of the mouse. Nat. Neurosci..

[bib4] Amaral D.G., Cowan W.M. (1980). Subcortical afferents to the hippocampal formation in the monkey. J. Comp. Neurol..

[bib5] Beier K.T., Saunders A., Oldenburg I.A., Miyamichi K., Akhtar N., Luo L., Whelan S.P.J., Sabatini B., Cepko C.L. (2011). Anterograde or retrograde transsynaptic labeling of CNS neurons with vesicular stomatitis virus vectors. Proc. Natl. Acad. Sci. USA.

[bib6] Beier K.T., Steinberg E.E., DeLoach K.E., Xie S., Miyamichi K., Schwarz L., Gao X.J., Kremer E.J., Malenka R.C., Luo L. (2015). Circuit Architecture of VTA Dopamine Neurons Revealed by Systematic Input-Output Mapping. Cell.

[bib7] Beier K.T., Kim C.K., Hoerbelt P., Hung L.W., Heifets B.D., DeLoach K.E., Mosca T.J., Neuner S., Deisseroth K., Luo L., Malenka R.C. (2017). Rabies screen reveals GPe control of cocaine-triggered plasticity. Nature.

[bib8] Beier K.T., Gao X.J., Xie S., DeLoach K.E., Malenka R.C., Luo L. (2019). Topological Organization of Ventral Tegmental Area Connectivity Revealed by Viral-Genetic Dissection of Input-Output Relations. Cell Rep..

[bib9] Bienkowski M.S., Bowman I., Song M.Y., Gou L., Ard T., Cotter K., Zhu M., Benavidez N.L., Yamashita S., Abu-Jaber J. (2018). Integration of gene expression and brain-wide connectivity reveals the multiscale organization of mouse hippocampal networks. Nat. Neurosci..

[bib10] Cembrowski M.S., Bachman J.L., Wang L., Sugino K., Shields B.C., Spruston N. (2016). Spatial Gene-Expression Gradients Underlie Prominent Heterogeneity of CA1 Pyramidal Neurons. Neuron.

[bib11] Cembrowski M.S., Phillips M.G., DiLisio S.F., Shields B.C., Winnubst J., Chandrashekar J., Bas E., Spruston N. (2018). Dissociable Structural and Functional Hippocampal Outputs via Distinct Subiculum Cell Classes. Cell.

[bib12] Cembrowski M.S., Wang L., Lemire A.L., Copeland M., DiLisio S.F., Clements J., Spruston N. (2018). The subiculum is a patchwork of discrete subregions. eLife.

[bib13] Cetin A., Komai S., Eliava M., Seeburg P.H., Osten P. (2006). Stereotaxic gene delivery in the rodent brain. Nat. Protoc..

[bib14] Chatterjee S., Sullivan H.A., MacLennan B.J., Xu R., Hou Y., Lavin T.K., Lea N.E., Michalski J.E., Babcock K.R., Dietrich S. (2018). Nontoxic, double-deletion-mutant rabies viral vectors for retrograde targeting of projection neurons. Nat. Neurosci..

[bib15] Choi K., Holly E.N., Davatolhagh M.F., Beier K.T., Fuccillo M.V. (2019). Integrated anatomical and physiological mapping of striatal afferent projections. Eur. J. Neurosci..

[bib16] Ciabatti E., González-Rueda A., Mariotti L., Morgese F., Tripodi M. (2017). Life-Long Genetic and Functional Access to Neural Circuits Using Self-Inactivating Rabies Virus. Cell.

[bib17] Ciocchi S., Passecker J., Malagon-Vina H., Mikus N., Klausberger T. (2015). Brain computation. Selective information routing by ventral hippocampal CA1 projection neurons. Science.

[bib18] Do J.P., Xu M., Lee S.-H., Chang W.-C., Zhang S., Chung S., Yung T.J., Fan J.L., Miyamichi K., Luo L., Dan Y. (2016). Cell type-specific long-range connections of basal forebrain circuit. eLife.

[bib19] Dolleman-van der Weel M.J., Griffin A.L., Ito H.T., Shapiro M.L., Witter M.P., Vertes R.P., Allen T.A. (2019). The nucleus reuniens of the thalamus sits at the nexus of a hippocampus and medial prefrontal cortex circuit enabling memory and behavior. Learn. Mem..

[bib20] Fürth D., Vaissière T., Tzortzi O., Xuan Y., Märtin A., Lazaridis I., Spigolon G., Fisone G., Tomer R., Deisseroth K. (2018). An interactive framework for whole-brain maps at cellular resolution. Nat. Neurosci..

[bib21] Ito H.T., Zhang S.-J., Witter M.P., Moser E.I., Moser M.-B. (2015). A prefrontal-thalamo-hippocampal circuit for goal-directed spatial navigation. Nature.

[bib22] Jimenez J.C., Su K., Goldberg A.R., Luna V.M., Biane J.S., Ordek G., Zhou P., Ong S.K., Wright M.A., Zweifel L. (2018). Anxiety Cells in a Hippocampal-Hypothalamic Circuit. Neuron.

[bib23] Kim Y., Spruston N. (2012). Target-specific output patterns are predicted by the distribution of regular-spiking and bursting pyramidal neurons in the subiculum. Hippocampus.

[bib24] Kim E.J., Jacobs M.W., Ito-Cole T., Callaway E.M. (2016). Improved Monosynaptic Neural Circuit Tracing Using Engineered Rabies Virus Glycoproteins. Cell Rep..

[bib25] Knierim J.J., Neunuebel J.P., Deshmukh S.S. (2013). Functional correlates of the lateral and medial entorhinal cortex: objects, path integration and local-global reference frames. Philos. Trans. R. Soc. Lond. B Biol. Sci..

[bib26] Kohara K., Pignatelli M., Rivest A.J., Jung H.-Y., Kitamura T., Suh J., Frank D., Kajikawa K., Mise N., Obata Y. (2014). Cell type-specific genetic and optogenetic tools reveal hippocampal CA2 circuits. Nat. Neurosci..

[bib27] Lerner T.N., Shilyansky C., Davidson T.J., Evans K.E., Beier K.T., Zalocusky K.A., Crow A.K., Malenka R.C., Luo L., Tomer R., Deisseroth K. (2015). Intact-Brain Analyses Reveal Distinct Information Carried by SNc Dopamine Subcircuits. Cell.

[bib28] Luo L., Callaway E.M., Svoboda K. (2018). Genetic Dissection of Neural Circuits: A Decade of Progress. Neuron.

[bib29] MacAskill A.F., Cassel J.M., Carter A.G. (2014). Cocaine exposure reorganizes cell type- and input-specific connectivity in the nucleus accumbens. Nat. Neurosci..

[bib30] Masurkar A.V., Srinivas K.V., Brann D.H., Warren R., Lowes D.C., Siegelbaum S.A. (2017). Medial and Lateral Entorhinal Cortex Differentially Excite Deep versus Superficial CA1 Pyramidal Neurons. Cell Rep..

[bib31] McHenry J.A., Otis J.M., Rossi M.A., Robinson J.E., Kosyk O., Miller N.W., McElligott Z.A., Budygin E.A., Rubinow D.R., Stuber G.D. (2017). Hormonal gain control of a medial preoptic area social reward circuit. Nat. Neurosci..

[bib32] Naber P.A., Witter M.P. (1998). Subicular efferents are organized mostly as parallel projections: a double-labeling, retrograde-tracing study in the rat. J. Comp. Neurol..

[bib33] Oh S.W., Harris J.A., Ng L., Winslow B., Cain N., Mihalas S., Wang Q., Lau C., Kuan L., Henry A.M. (2014). A mesoscale connectome of the mouse brain. Nature.

[bib34] O’Keefe J., Dostrovsky J. (1971). The hippocampus as a spatial map. Preliminary evidence from unit activity in the freely-moving rat. Brain Res..

[bib35] Okuyama T., Kitamura T., Roy D.S., Itohara S., Tonegawa S. (2016). Ventral CA1 neurons store social memory. Science.

[bib36] Reardon T.R., Murray A.J., Turi G.F., Wirblich C., Croce K.R., Schnell M.J., Jessell T.M., Losonczy A. (2016). Rabies Virus CVS-N2c(ΔG) Strain Enhances Retrograde Synaptic Transfer and Neuronal Viability. Neuron.

[bib37] Ren J., Friedmann D., Xiong J., Liu C.D., Ferguson B.R., Weerakkody T., DeLoach K.E., Ran C., Pun A., Sun Y. (2018). Anatomically Defined and Functionally Distinct Dorsal Raphe Serotonin Sub-systems. Cell.

[bib38] Roy D.S., Kitamura T., Okuyama T., Ogawa S.K., Sun C., Obata Y., Yoshiki A., Tonegawa S. (2017). Distinct Neural Circuits for the Formation and Retrieval of Episodic Memories. Cell.

[bib39] Schwarz L.A., Miyamichi K., Gao X.J., Beier K.T., Weissbourd B., DeLoach K.E., Ren J., Ibanes S., Malenka R.C., Kremer E.J., Luo L. (2015). Viral-genetic tracing of the input-output organization of a central noradrenaline circuit. Nature.

[bib40] Smith J.B., Klug J.R., Ross D.L., Howard C.D., Hollon N.G., Ko V.I., Hoffman H., Callaway E.M., Gerfen C.R., Jin X. (2016). Genetic-Based Dissection Unveils the Inputs and Outputs of Striatal Patch and Matrix Compartments. Neuron.

[bib41] Soltesz I., Losonczy A. (2018). CA1 pyramidal cell diversity enabling parallel information processing in the hippocampus. Nat. Neurosci..

[bib42] Strange B.A., Witter M.P., Lein E.S., Moser E.I. (2014). Functional organization of the hippocampal longitudinal axis. Nat. Rev. Neurosci..

[bib43] Sun Y., Nguyen A.Q., Nguyen J.P., Le L., Saur D., Choi J., Callaway E.M., Xu X. (2014). Cell-type-specific circuit connectivity of hippocampal CA1 revealed through Cre-dependent rabies tracing. Cell Rep..

[bib44] Sun Q., Li X., Ren M., Zhao M., Zhong Q., Ren Y., Luo P., Ni H., Zhang X., Zhang C. (2019). A whole-brain map of long-range inputs to GABAergic interneurons in the mouse medial prefrontal cortex. Nat. Neurosci..

[bib45] Tervo D.G.R., Hwang B.-Y., Viswanathan S., Gaj T., Lavzin M., Ritola K.D., Lindo S., Michael S., Kuleshova E., Ojala D. (2016). A Designer AAV Variant Permits Efficient Retrograde Access to Projection Neurons. Neuron.

[bib46] van Groen T., Miettinen P., Kadish I. (2003). The entorhinal cortex of the mouse: organization of the projection to the hippocampal formation. Hippocampus.

[bib47] Vertes R.P. (2006). Interactions among the medial prefrontal cortex, hippocampus and midline thalamus in emotional and cognitive processing in the rat. Neuroscience.

[bib48] Wall N.R., De La Parra M., Callaway E.M., Kreitzer A.C. (2013). Differential innervation of direct- and indirect-pathway striatal projection neurons. Neuron.

[bib49] Weissbourd B., Ren J., DeLoach K.E., Guenthner C.J., Miyamichi K., Luo L. (2014). Presynaptic partners of dorsal raphe serotonergic and GABAergic neurons. Neuron.

[bib50] Winnubst J., Bas E., Ferreira T.A., Wu Z., Economo M.N., Edson P., Arthur B.J., Bruns C., Rokicki K., Schauder D. (2019). Reconstruction of 1,000 Projection Neurons Reveals New Cell Types and Organization of Long-Range Connectivity in the Mouse Brain. Cell.

[bib51] Wyss J.M., Swanson L.W., Cowan W.M. (1979). A study of subcortical afferents to the hippocampal formation in the rat. Neuroscience.

[bib52] Xu W., Südhof T.C. (2013). A neural circuit for memory specificity and generalization. Science.

