# Supplementary material for: Biased Connectivity of Brain-wide Inputs to Ventral Subiculum Output Neurons
Source: Cell Rep. 2020 Mar 17;30(11):3644–3654.e6. doi: 10.1016/j.celrep.2020.02.093 (PMC7090382; doi:10.1016/j.celrep.2020.02.093)
Supplement: Document S1. Figures S1–S4 and Tables S1 and S2 [file mmc1.pdf]

Cell Reports, Volume *30*

Supplemental Information

Biased Connectivity of Brain-wide Inputs to Ventral Subiculum Output Neurons

Ryan W.S. Wee and Andrew F. MacAskill

Biased connectivity of brain-wide inputs to ventral subiculum output neurons

Ryan W.S. Wee and Andrew F. MacAskill *
Department of Neuroscience, Physiology and Pharmacology, University College London, Gower St, London, WC1E 6BT
*Lead contact: a.macaskill@ucl.ac.uk

Supplemental Information

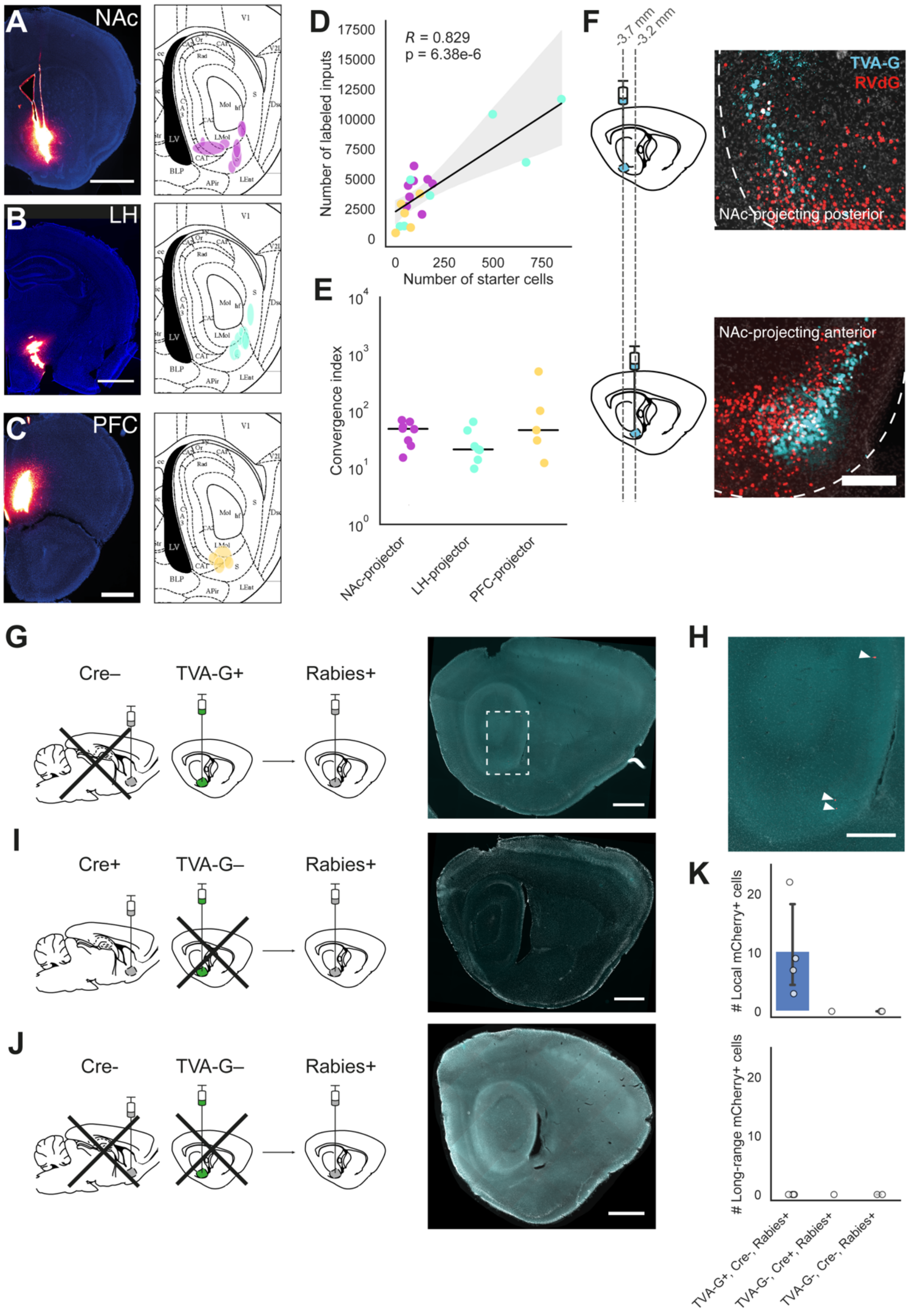
A
NAc
B
LH
C
PFC
D
R = 0.829
p = 6.38e-6
Number of labeled inputs
17500
15000
12500
10000
7500
5000
2500
0
0
250
500
750
Number of starter cells
E
Convergence index
10^4
10^3
10^2
10^1
10^0
NAc-projector
LH-projector
PFC-projector
F
-3.7 mm
-3.2 mm
TVA-G
RVdG
NAc-projecting posterior
NAc-projecting anterior
G
Cre–
TVA-G+
Rabies+
H
I
Cre+
TVA-G–
Rabies+
J
Cre-
TVA-G–
Rabies+
K
Local mCherry+ cells
20
10
0
Long-range mCherry+ cells
20
10
0
TVA-G+, Cre-, Rabies+
TVA-G-, Cre+, Rabies+
TVA-G-, Cre-, Rabies+

Figure S1: TRIO starter cell quantification and experimental controls. Related to Figure 2.

(A – C) *Right:* Example stitched images of injection sites using identical volumes of CTXβ-647 and stereotactic coordinates as for *AAV2-retro-Cre* injections. All injection sites were localised in the targeted regions. Scale bar: 1000 µm (A–B), 500 µm. *Left:* Starter cell centre-of-mass (COM) plotted onto a reference plate from the Paxinos atlas for each vS projection; each ellipse represents the starter cell geometric mean from one brain sample (horizontal and vertical widths represent 1 s.d. about the mean coordinate position).

(D) Scatter plot of the number of starter cells against the total number of rabies-labelled inputs counted in each brain sample. The number of labelled inputs scales with the number of starter cells (Pearson correlation, $R^2 = 0.829$, $p = 6.38 \times 10^{-6}$). Shaded regions represent bootstrapped 95% confidence intervals.

(E) Convergent indices (the total number of inputs divided by the number of starter cells) plotted for each projection population. The solid line indicates the median convergent index.

(F) *Left:* Schematic to control for starter cell location by injecting TVA-G and rabies into either a more posterior (AP position: -3.7 mm) or anterior position (AP position: -3.2 mm). *Right:* Stitched images of posterior (top) and anterior (bottom) vS, with starter cell labelling. This strategy allowed us to isolate vS^{NAc} starter cells across different COM positions (see **Figure 3B**). Scale bar: 200 µm.

(G, I, J) Schematics for control surgeries for TRIO. Example stitched images of sagittal sections are shown on the right.

(G, H) Controls with injection of the single-construct TVA-G virus into vS without *AAV2-retro-Cre* injection in the output side. Rabies virus was injected 2 weeks later (n = 4 brains). (G) *Right:* Sagittal brain section from a representative experiment without Cre injection. (H) Zoom-in image of boxed region in (G, right). Arrowheads indicate sparse mCherry+ rabies-labelled cells, likely due to Cre-independent expression of TVA-G and subsequent rabies infection of starter cells.

(I, J) No TVA-G (I, n = 1 brain) and no TVA-G and Cre (J, n = 2 brains) controls. No mCherry+ cells were detected in (I) and (J).

(K) Quantification of mCherry+ labelled inputs in control conditions. All leaky mCherry+ cells were detected locally within the hippocampal formation, and none were detected in long-range input regions. Bar plots indicate mean ± sem. Scale bars: 1000 µm (G,I,J, *Right*), 500 µm (H, zoomed-in image)

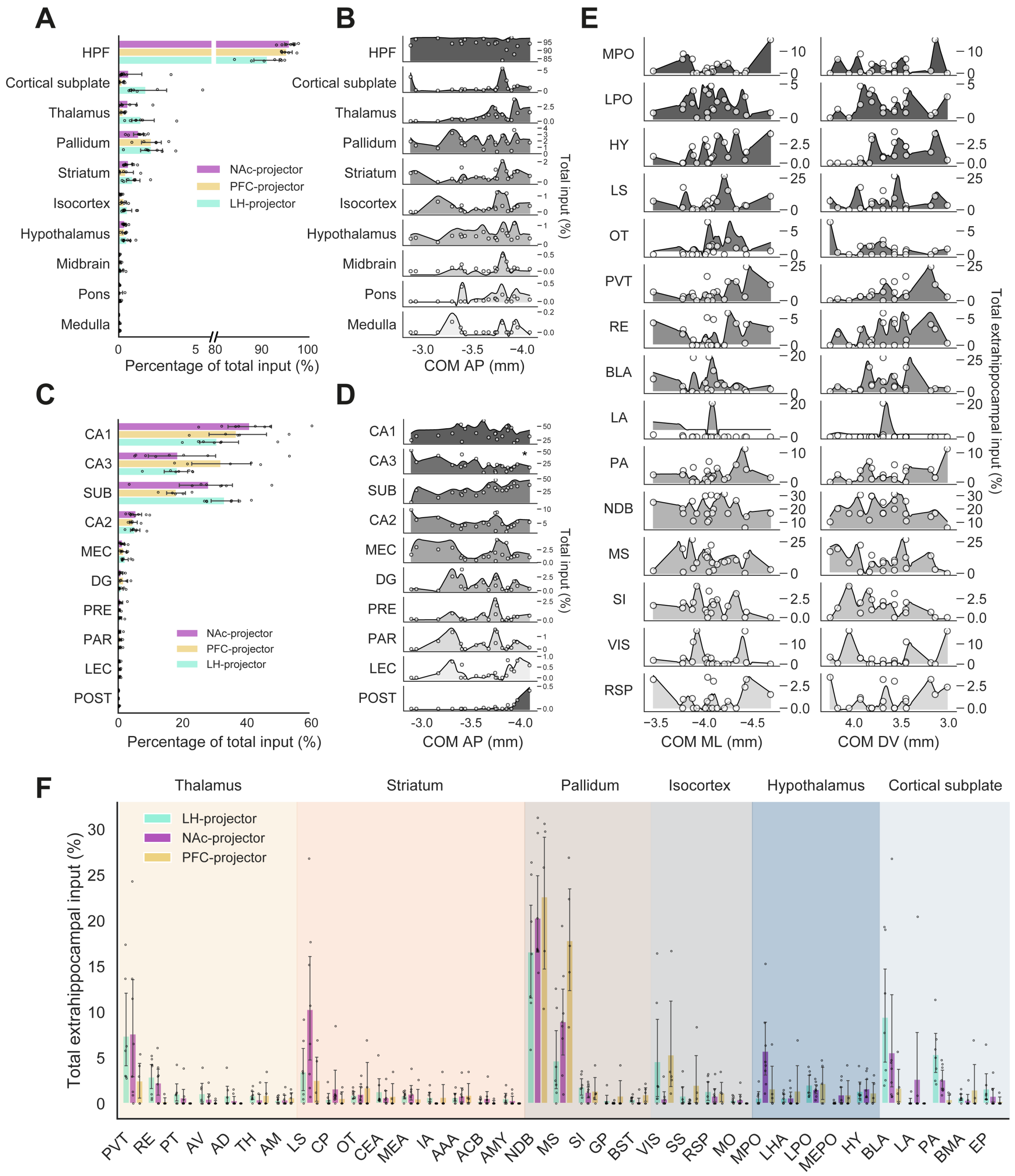
A
HPF
Cortical subplate
Thalamus
Pallidum
Striatum
Isocortex
Hypothalamus
Midbrain
Pons
Medulla
NAc-projector
PFC-projector
LH-projector
0
5
80
90
100
Percentage of total input (%)
B
-3.0
-3.5
-4.0
COM AP (mm)
Total input (%)
E
MPO
LPO
HY
LS
OT
PVT
RE
BLA
LA
PA
NDB
MS
SI
VIS
RSP
COM ML (mm)
COM DV (mm)
Total extrahippocampal input (%)
C
CA1
CA3
SUB
CA2
MEC
DG
PRE
PAR
LEC
POST
0
20
40
60
Percentage of total input (%)
D
F
Thalamus
Striatum
Pallidum
Isocortex
Hypothalamus
Cortical subplate
Total extrahippocampal input (%)
PVT
RE
PT
AV
AD
TH
AM
LS
CP
OT
CEA
MEA
IA
AAA
ACB
AMY
NDB
MS
SI
GP
BST
VIS
SS
RSP
MO
MPO
LHA
LPO
MEPO
HY
BLA
LA
PA
BMA
EP

Figure S2: Coarse, intra- and extrahippocampal input mapping. Related to Figure 3.

(A) Coarse-level rabies-labelled inputs split by output projection and normalised to the total number of inputs counted within a single brain. HPF: Hippocampal formation. Bar plots indicate mean ± sem.

(B) Same dataset as in A but plotted as a function of COM AP coordinates. The shaded continuous distribution represents the smoothed input density (normalised with area under the curve = 1) as a function of COM.

(C) Intrahippocampal inputs split according to output projection. No intrahippocampal input fractions were significantly different across projection populations after either multiple linear regression with ANOVA analysis or multiple one-way ANOVA comparisons across projection populations (see Extended Table 2).

(D) Same dataset as in C but plotted as a function of COM AP coordinates. CA3 input fraction (*$p < 0.05$) was detected as having statistically significant model fit (see Extended Table 2).

(E) The input fractions are plotted as a function of COM along the ML (*Left*) and DV (*Right*) axes. The shaded continuous line represents the smoothed input density.

(F) Data for long-range inputs from 35 extrahippocampal brain regions by each projection population. Note that the dataset was preprocessed by including only input regions that generated at least 0.25% of extrahippocampal input. Data expressed as a percentage of total extrahippocampal input cells counted in a single brain sample. Abbreviations: see Extended Table 1.

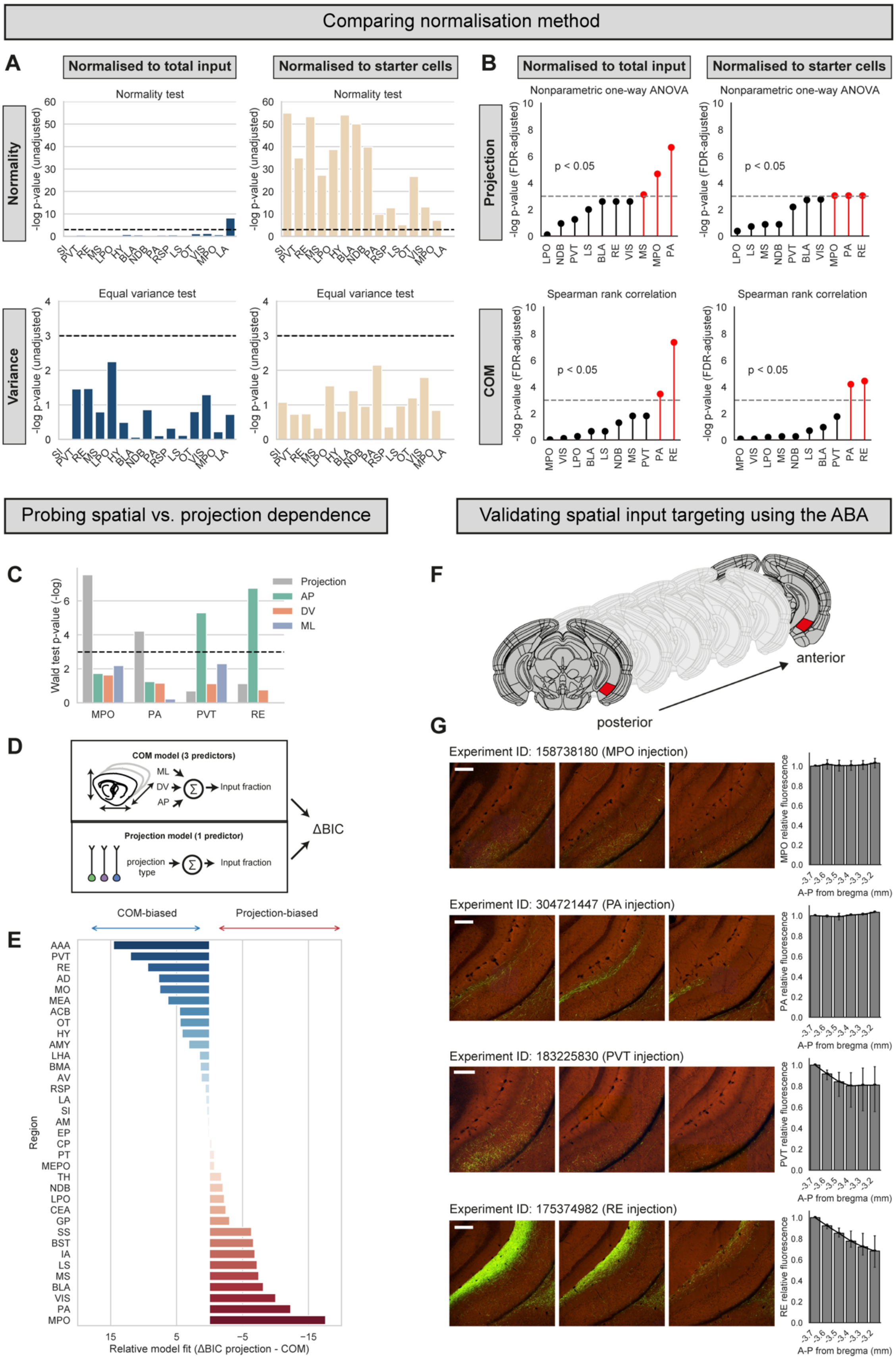
Comparing normalisation method
A
Normalised to total input
Normalised to starter cells
Normality
Normality test
-log p-value (unadjusted)
Variance
Equal variance test
B
Nonparametric one-way ANOVA
Projection
-log p-value (FDR-adjusted)
p < 0.05
COM
Spearman rank correlation
Probing spatial vs. projection dependence
C
Projection
AP
DV
ML
Wald test p-value (-log)
MPO
PA
PVT
RE
D
COM model (3 predictors)
Input fraction
Projection model (1 predictor)
projection type
ΔBIC
E
COM-biased
Projection-biased
Region
Relative model fit (ΔBIC projection - COM)
Validating spatial input targeting using the ABA
F
anterior
posterior
G
Experiment ID: 158738180 (MPO injection)
MPO relative fluorescence
A-P from bregma (mm)
Experiment ID: 304721447 (PA injection)
PA relative fluorescence
Experiment ID: 183225830 (PVT injection)
PVT relative fluorescence
Experiment ID: 175374982 (RE injection)
RE relative fluorescence

Figure S3: Spatial and projection dependence analysis of rabies-labelled inputs. Related to Figure 3.

(A) Control analysis to compare normalisation methods to either extrahippocampal total input (*Left*) or number of starter cells (*Right*). Normality was assessed using the Jarque-Bera test, and the presence of heteroscedasticity was assessed using Levene's test. Normalising to starter cells creates non-normal input distributions for most inputs compared to normalising to total inputs. Both normalisation methods produce homoscedastic distributions. Note that both datasets have been log transformed prior to testing the normality and variance of the datasets.

(B) Nonparametric statistical testing was applied to compare the two normalisation methods, given the non-normality of the dataset normalised to the number of starter cells. *Left:* multiple nonparametric Kruskal-Wallis one-way ANOVA (adjusted for multiple comparisons with the Benjamini-Hochberg method with FDR < 0.05) reveals significantly different inputs across projection population; multiple Spearman rank correlation tests reveal inputs that significantly differ across COM AP. This was common to both normalisation methods: after normalising to total input (*Left*) or to number of starter cells (*Right*). Note that statistical tests were conducted only on the top ten input regions.

(C) After fitting full models, the statistical significance of each predictor was tested using the Wald test. MPO and PA are highly projection-specific, where only the projection type predictor was significant. By contrast, PVT and RE inputs are highly spatially dependent, where the COM along the AP axis was significant.

(D) Schematic of single-predictor linear models where COM in all 3 brain axes of starter cells or projection identity were used as predictors, and the input fraction normalised to total input was used as the target variable for each brain region. The two competing models were then compared with ΔBIC as measures of goodness-of-fit.

(E) For each of 35 brain regions that provide input to vS, the Bayesian information criterion (BIC) score was calculated for the COM and projection model, and the difference between the BIC scores of the competing models were plotted as a measure of goodness-of-fit. Positive ΔBIC values indicate that a given input is biased towards COM, while negative ΔBIC values indicate an input biased towards projection type.

(F) Analysis of anterograde tracing experiments from the ABA. Six coronal sections, spaced 100 µm apart and covering the ventral subiculum region, were downloaded from the ABA and analysed. The region shaded in red are the approximate ROIs which were used to compute the relative intensity of axonal projections.

(G) *Left:* Example consecutive image series (depicting every second section of the six sections) for each experiment showing the ventral subiculum region. Each image is a zoom-in from a stitched image downloaded from the Allen Brain Atlas. Scale bar: 100 µm. *Right:* Relative fluorescence intensity of axonal projections in the ventral subiculum, normalised to the posterior-most section. MPO and PA injection experiments (n = 3 and n = 2 brains, respectively) show consistent axonal projection density across the six sections, while the RE and PVT injection experiments (n = 3 brains each) display prominent spatial targeting along the AP axis. Error bars: 500 µm. Bar plots represent mean ± sem.

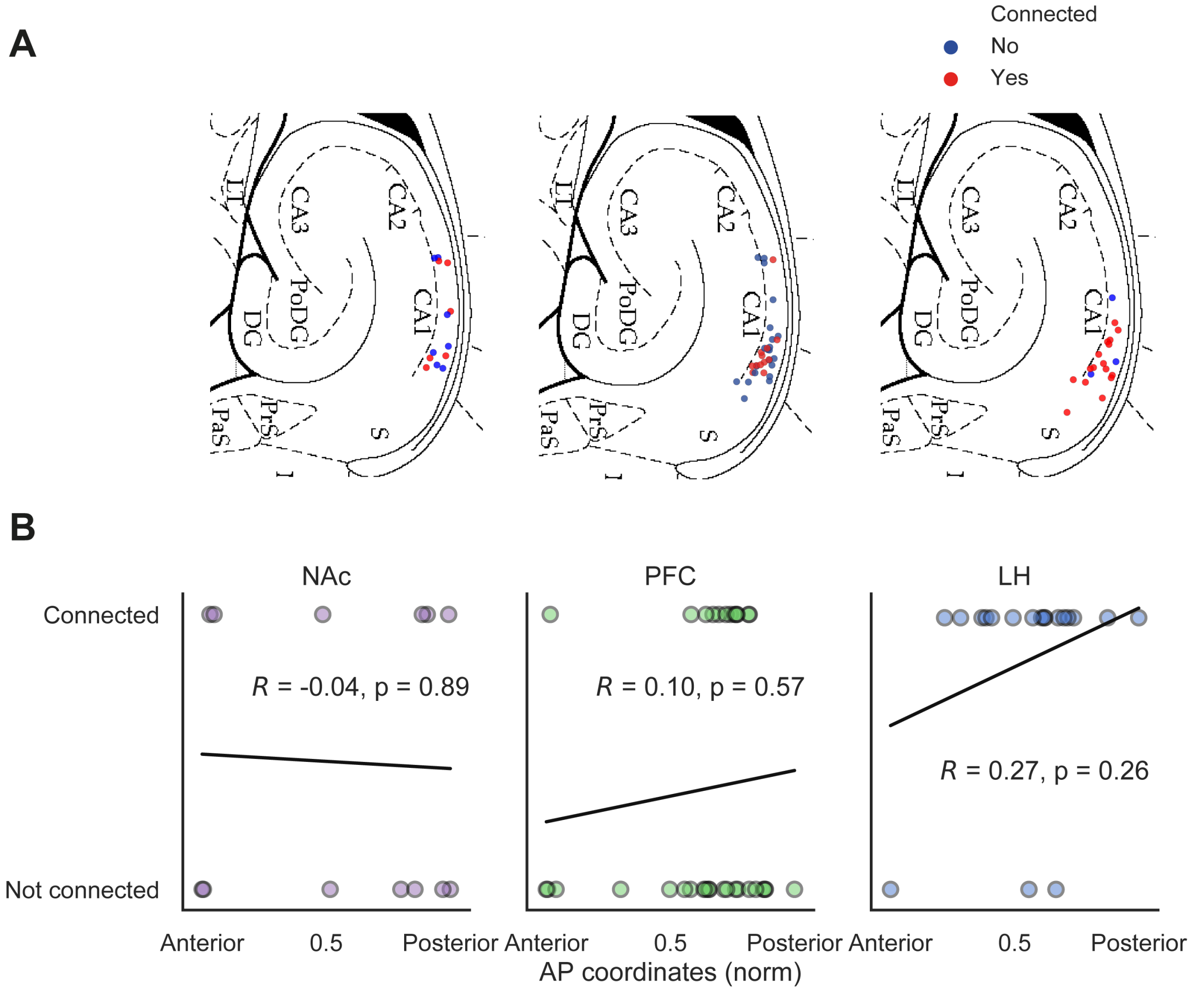
A
Connected
No
Yes
CA3
CA2
CA1
PoDG
DG
LI
PaS
PrS
S
B
NAc
PFC
LH
Connected
Not connected
R = -0.04, p = 0.89
R = 0.10, p = 0.57
R = 0.27, p = 0.26
Anterior
0.5
Posterior
AP coordinates (norm)

Figure S4: RE input connectivity by projection identity. Related to Figure 4.

(A) Map of RE input connectivity split by projection population. Each dot represents a patched cell, and cells were considered connected with RE input if the light-evoked photo response exceeds > 5 pA. Diagram of map taken from the Paxinos atlas.

(B) Input connectivity split by the projection identity of patched neurons and plotted against AP coordinates. For all projection populations, there was no statistically significant correlation between the AP coordinate and RE connection probability of cells.

Extended Table 1. Related to STAR Methods.

Abbreviation	Brain region
PVT	Paraventricular nucleus of the thalamus
RE	Nucleus of reuniens
AV	Anteroventral nucleus of thalamus
AD	Anterodorsal nucleus
PT	Parataenial nucleus
TH	Thalamus
CL	Central lateral nucleus of the thalamus
AM	Anteromedial nucleus
LS	Lateral septal nucleus
CP	Caudoputamen
OT	Olfactory tubercle
CEA	Central amygdalar nucleus
MEA	Medial amygdalar nucleus
IA	Intercalated amygdalar nucleus
AAA	Anterior amygdalar area
ACB	Nucleus accumbens
AMY	Unannotated amygdala
P	Pons
NDB	Diagonal band nucleus
MS	Medial septal nucleus
SI	Substantia innominata
GP	Globus pallidus
BST	Bed nuclei of the stria terminalis
APN	Anterior pretectal nucleus
VIS	Visual areas
SS	Somatosensory areas
RSP	Retrosplenial area
MO	Somatomotor areas
MPO	Medial preoptic area
LHA	Lateral hypothalamic area
LPO	Lateral preoptic area
MEPO	Median preoptic nucleus
HY	Hypothalamus
BLA	Basolateral amygdalar nucleus
LA	Lateral amygdalar nucleus
PA	Posterior amygdalar nucleus
BMA	Basomedial amygdalar nucleus
EP	Endopiriform nucleus
CA1	Field cornu ammonis 1
CA2	Field cornu ammonis 2
CA3	Field cornu ammonis 3
SUB	Subiculum
DG	Dentate gyrus
LEC	Lateral entorhinal cortex
MEC	Medial entorhinal cortex
PRE	Presubiculum
PARA	Parasubiculum

Extended Table 2: Statistical summary. Related to STAR Methods

FIGURE	DESCRIPTORS	N	TEST USED	TEST STATISTIC	P VALUE
Fig 1F (*Left*)	AP<br>ML<br>DV<br>Pearson correlation coefficient of coronally sectioned data	n = 10 hemispheres from 6 animals	**Repeated-measures one-way ANOVA**<br><br>**Post-hoc two-tailed paired t-test with Benjamini-Hochberg (BH) correction**<br><br>AP vs. ML<br>AP vs. DV<br>DV vs. ML	$F_{2,18}$ = 12.102<br><br><br><br>t(9) = 3.573<br>t(9) = 4.196<br>t(9) = -1.019	p = 0.0005<br><br><br><br>p = 0.009<br>p = 0.007<br>p = 0.335
Fig 1F (*Right*)	AP<br>DV<br>ML<br>10-fold cross-validated Δ accuracy of logistic regression models of coronally sectioned data	n = 10 hemispheres from 6 animals	**Repeated-measures one-way ANOVA**<br><br>**Post-hoc two-tailed paired t-test with BH correction**<br>AP vs. ML<br>AP vs. DV<br>DV vs. ML	$F_{2,18}$ = 7.698<br><br><br>t(9) = -3.109<br>t(9) = -2.648<br>t(9) = 0.457	p = 0.0038<br><br><br>p = 0.0398<br>p = 0.0376<br>p = 0.6584
Fig 1J (*Left*)	PD<br>DV<br>DS<br>Pearson correlation coefficient of horizontally sectioned data (PD axis)	n = 10 hemispheres from 7 animals	**Repeated-measures one-way ANOVA**<br><br>**Post-hoc two-tailed paired t-test with BH correction**<br>PD vs. DV<br>DS vs. PD<br>DS vs. DV	$F_{2,18}$ = 49.214<br><br><br>t(9) = -12.393<br>t(9) = -7.855<br>t(9) = 1.149	p = 0.0000<br><br><br>p = 1.75e-6<br>p = 3.84e-5<br>p = 0.280
Fig 1J (*Right*)	PD<br>DV<br>DS<br>10-fold cross-validated Δ accuracy of logistic regression models of horizontally sectioned data	n = 10 hemispheres from 7 animals	**Repeated-measures one-way ANOVA**<br><br>**Post-hoc two-tailed paired t-test with BH correction**<br>DS vs. DV<br>DS vs. PD<br>DV vs. PD	$F_{2,18}$ = 10.829<br><br><br>t(9) = -1.504<br>t(9) = 3.067<br>t(9) = 3.667	p = 0.0008<br><br><br>p = 0.167<br>p = 0.0201<br>p = 0.0167
Fig 3D	Dependent variable: input normalised to extrahippocampal input<br><br>Independent variables: COM and projection	n = 20 brains 15 input regions	**Multiple linear regression** model fits assessed with ANOVA (p-values adjusted with the Benjamini-Hochberg method, FDR < 0.05)	BLA : F-statistic = 2.772<br>HY : F-statistic = 1.297<br>LA : F-statistic = 2.202<br>LPO : F-statistic = 0.165<br>LS : F-statistic = 1.185<br>MPO : F-statistic = 5.502<br>MS : F-statistic = 1.906<br>NDB : F-statistic = 0.701<br>OT : F-statistic = 2.083<br>PA : F-statistic = 6.424<br>PVT : F-statistic = 4.981<br>RE : F-statistic = 6.023<br>RSP : F-statistic = 1.312<br>SI : F-statistic = 1.171<br>VIS : F-statistic = 1.534<br><br>For all tests, residual df = 14, model df = 5	BLA, p = 0.182<br>HY, p = 0.428<br>LA, p = 0.274<br>LPO, p = 0.971<br>LS, p = 0.428<br>MPO, p = 0.026<br>MS, p = 0.294<br>NDB, p = 0.677<br>OT, p = 0.274<br>PA, p = 0.026<br>PVT, p = 0.030<br>RE, p = 0.026<br>RSP, p = 0.428<br>SI, p = 0.428<br>VIS, p = 0.403
Fig 3E	Full (COM and projection models) vs. single-predictor COM (COM-only models), projection (projection-only models)	20 brains (observations) per input region, per model fitted	**Likelihood ratio tests**<br><br>COM vs. full model (p-values adjusted with the Benjamini-Hochberg method, FDR < 0.05)	 <br>BLA, χ²(1) = 11.611<br>HY, χ²(1) = 0.093<br>LA, χ²(1) = 6.340<br>LPO, χ²(1) = 0.135<br>LS, χ²(1) = 5.728<br>MPO, χ²(1) = 21.547<br>MS, χ²(1) = 4.669<br>NDB, χ²(1) = 1.052<br>OT, χ²(1) = 3.609<br>PA, χ²(1) = 12.047	 <br>BLA, p = 0.015<br>HY, p = 0.955<br>LA, p = 0.126<br>LPO, p = 0.955<br>LS, p = 0.143<br>MPO, p = 0.000<br>MS, p = 0.208<br>NDB, p = 0.682<br>OT, p = 0.288<br>PA, p = 0.015

				PVT, χ²(1) = 1.991	PVT, p = 0.462
				RE, χ²(1) = 3.208	RE, p = 0.302
				RSP, χ²(1) = 3.514	RSP, p = 0.288
				SI, χ²(1) = 2.709	SI, p = 0.352
				VIS, χ²(1) = 7.609	VIS, p = 0.084
			Projection vs. full models (p-values adjusted with the Benjamini-Hochberg method, FDR < 0.05)	BLA, χ²(1) = 6.446	BLA, p = 0.172
				HY, χ²(1) = 7.239	HY, p = 0.158
				LA, χ²(1) = 9.903	LA, p = 0.073
				LPO, χ²(1) = 0.896	LPO, p = 0.954
				LS, χ²(1) = 1.519	LS, p = 0.847
				MPO, χ²(1) = 6.950	MPO, p = 0.158
				MS, χ²(1) = 0.219	MS, p = 0.974
				NDB, χ²(1) = 1.994	NDB, p = 0.782
				OT, χ²(1) = 11.079	OT, p = 0.057
				PA, χ²(1) = 2.732	PA, p = 0.652
				PVT, χ²(1) = 16.968	PVT, p = 0.010
				RE, χ²(1) = 15.598	RE, p = 0.010
				RSP, χ²(1) = 7.168	RSP, p = 0.158
				SI, χ²(1) = 6.138	SI, p = 0.175
				VIS, χ²(1) = 0.575	VIS, p = 0.967
Fig 3F	Total extrahippocampal fraction (log transformed) ± sem	20 brains 15 input regions	**Multiple one-way ANOVAs** (p-values adjusted with the Benjamini-Hochberg method, FDR < 0.05)	BLA, F-statistic = 3.755	BLA, p = 0.112
				HY, F-statistic = 0.159	HY, p = 0.965
				LA, F-statistic = 0.755	LA, p = 0.728
				LPO, F-statistic = 0.106	LPO, p = 0.965
				LS, F-statistic = 2.713	LS, p = 0.203
				MPO, F-statistic = 9.304	MPO, p = 0.014
				MS, F-statistic = 5.631	MS, p = 0.066
				NDB, F-statistic = 1.120	NDB, p = 0.582
				OT, F-statistic = 0.018	OT, p = 0.982
				PA, F-statistic = 15.925	PA, p = 0.002
				PVT, F-statistic = 1.612	PVT, p = 0.428
				RE, F-statistic = 3.779	RE, p = 0.112
				RSP, F-statistic = 0.223	RSP, p = 0.965
				SI, F-statistic = 0.368	SI, p = 0.951
				VIS, F-statistic = 4.284	VIS, p = 0.112
				For all tests, residual df = 14, model df = 5	
			**Post-hoc Tukey**		
			RE: vS^{LH} and vS^{PFC}	q = -1.052 [-2.056, -0.048]	p < 0.05
			RE: vS^{LH} and vS^{NAc}	q = -0.261 [-1.149, 0.626]	p > 0.05
			RE: vS^{NAc} and vS^{PFC}	q = -0.791 [-1.768, 0.187]	p > 0.05
			MPO: vS^{LH} and vS^{PFC}	q = 0.277 [-0.691, 1.246]	p > 0.05
			MPO: vS^{LH} and vS^{NAc}	q = 1.367 [0.511, 2.223]	p < 0.05
			MPO: vS^{NAc} and vS^{PFC}	q = -1.090 [-2.032, -0.147]	p < 0.05
			PA: vS^{LH} and vS^{PFC}	q = -1.455 [-2.117, -0.793]	p < 0.05
			PA: vS^{LH} and vS^{NAc}	q = -0.548 [-1.133, 0.0376]	p > 0.05
			PA: vS^{NAc} and vS^{PFC}	q = -0.907 [-1.552, -0.263]	p < 0.05
			PVT: vS^{LH} and vS^{PFC}	q = -0.966 [-2.365, 0.433]	p > 0.05
			PVT: vS^{LH} and vS^{NAc}	q = -0.277 [-1.513, 0.960]	p > 0.05
			PVT: vS^{NAc} and vS^{PFC}	q = -0.689 [-2.051, 0.673]	p > 0.05
Fig 3G	Scatterplots of extrahippocampal input fractions (log transformed) as a function of COM along the AP axis	20 brains 15 regions	**Multiple Spearman rank correlations** (COM vs. input fraction; p-values adjusted with the Benjamini-Hochberg method, FDR < 0.05)	BLA, R = -0.214	BLA, p = 0.683
				HY, R = 0.118	HY, p = 0.790
				LA, R = -0.097	LA, p = 0.790
				LPO, R = 0.123	LPO, p = 0.790
				LS, R = -0.218	LS, p = 0.683
				MPO, R = -0.008	MPO, p = 0.972
				MS, R = 0.424	MS, p = 0.245
				NDB, R = 0.345	NDB, p = 0.409
				OT, R = 0.177	OT, p = 0.760
				PA, R = -0.589	PA, p = 0.047
				PVT, R = -0.420	PVT, p = 0.245
				RE, R = -0.773	RE, p = 0.001
				RSP, R = -0.273	RSP, p = 0.612
				SI, R = -0.101	SI, p = 0.790
				VIS, R = -0.063	VIS, p = 0.850

Fig 4a	Non-vS^{PFC}<br>vS^{PFC}<br>median and interquartile range	11 brains<br>5 brains	**Mann-Whitney U test**	U = 12.0	p = 0.0129
Fig 4g	vS^{LH}<br>vS^{NAc}<br>median and 95% bootstrapped CI	18 cells<br>18 cells	**Wilcoxon signed-rank test**	V = 4.0	p = 3.86e-4
Fig 4k	vS^{NAc}<br>vS^{PFC}<br>median and 95% bootstrapped CI	11 cells<br>11 cells	**Wilcoxon signed-rank test**	V = 6.0	p = 0.0164
Fig S1D	Linear regression (number of starter cells regressed against total number of inputs)	16 brains	**Pearson correlation**	R = 0.829	p = 6.377e-6
Fig S2C–D	Dependent variable: input normalised to total input<br><br>Independent variables: COM and projection	20 brains<br>10 input regions	**Multiple linear regression** (p-values adjusted with the Benjamini-Hochberg method, FDR < 0.05)	CA1, F-statistic = 1.874<br>CA3, F-statistic = 6.405<br>SUB, F-statistic = 3.882<br>CA2, F-statistic = 1.187<br>MEC, F-statistic = 1.705<br>DG, F-statistic = 2.170<br>PRE, F-statistic = 2.807<br>PAR, F-statistic = 2.393<br>LEC, F-statistic = 2.180<br>POST, F-statistic = 2.671	CA1, p = 0.204<br>CA3, p = 0.027<br>SUB, p = 0.102<br>CA2, p = 0.364<br>MEC, p = 0.220<br>DG, p = 0.166<br>PRE, p = 0.166<br>PAR, p = 0.166<br>LEC, p = 0.166<br>POST, p = 0.166
Fig S2C	Total input fraction ± sem	20 brains<br>10 input regions	**Multiple one-way ANOVAs** (p-values adjusted with the Benjamini-Hochberg method, FDR < 0.05)	CA1, F-statistic = 1.984<br>CA2, F-statistic = 0.177<br>CA3, F-statistic = 2.554<br>DG-, F-statistic = 0.721<br>LEC, F-statistic = 0.404<br>MEC, F-statistic = 0.969<br>PAR, F-statistic = 0.341<br>POST, F-statistic = 2.125<br>PRE, F-statistic = 0.204<br>SUB, F-statistic = 2.104<br>For all tests, within group df = 16, between group df = 2	CA1, p = 0.420<br>CA2, p = 0.840<br>CA3, p = 0.420<br>DG-, p = 0.834<br>LEC, p = 0.840<br>MEC, p = 0.799<br>PAR, p = 0.840<br>POST, p = 0.420<br>PRE, p = 0.840<br>SUB, p = 0.420
Fig S2D	Scatterplots of extrahippocampal input fractions as a function of COM	20 brains<br>10 regions	**Multiple Spearman rank correlations** (COM vs. input fraction; p-values adjusted with the Benjamini-Hochberg method, FDR < 0.05)	CA1, R = 0.099<br>CA2, R = 0.208<br>CA3, R = 0.532<br>DG, R = -0.038<br>LEC, R = -0.424<br>MEC, R = 0.197<br>PAR, R = -0.179<br>POST, R = -0.631<br>PRE, R = -0.121<br>SUB, R = -0.795	CA1, p = 0.752<br>CA2, p = 0.644<br>CA3, p = 0.052<br>DG, p = 0.875<br>LEC, p = 0.156<br>MEC, p = 0.644<br>PAR, p = 0.644<br>POST, p = 0.014<br>PRE, p = 0.752<br>SUB, p = 0.000
Fig S3C	Dependent variable: input normalised to extrahippocampal input<br><br>Independent variables: COM and projection	20 brains<br>4 input region hits	**Wald test for model coefficients**<br><br>RE full model<br><br><br><br><br><br>PA full model<br><br><br><br><br>MPO full model<br><br><br><br><br><br>PVT full model	$W_{projection}$ = 1.218<br>W_{AP} = 16.453<br>W_{ML} = 0.552<br>W_{DV} = 0.00143<br><br><br>$W_{projection}$ = 5.785<br>W_{AP} = 1.222<br>W_{ML} = 1.085<br>W_{DV} = 0.0639<br><br>$W_{projection}$ = 13.558<br>W_{AP} = 2.013<br>W_{ML} = 1.853<br>W_{DV} = 2.874<br><br><br>$W_{projection}$ = 0.733<br>W_{AP} = 11.062<br>W_{ML} = 1.038<br>W_{DV} = 3.104	p = 0.325<br>p = 0.00118<br>p = 0.470<br>p = 0.970<br><br><br>p = 0.0148<br>p = 0.288<br>p = 0.315<br>p = 0.804<br><br>p = 5.31e-4<br>p = 0.178<br>p = 0.195<br>p = 0.112<br><br><br>p = 0.498<br>p = 0.005<br>p = 0.326<br>p = 0.0999

Fig S4B	Scatter plots of AP position (normalised) against RE input connectivity for vS^{NAc}, vS^{LH} and vS^{PFC}	vS^{NAc} = 13 cells<br>vS^{PFC} = 33 cells<br>vS^{LH} = 19 cells	**Point biserial correlation coefficients**	R_{NAc} = -0.04<br>R_{LH} =0.27<br>R_{PFC} = 0.10	p_{NAc} = 0.89<br>p_{LH} =0.26<br>p_{PFC} = 0.57
